# [^11^C]Carbon monoxide: advances in production and application to PET radiotracer development over the past 15 years

**DOI:** 10.1186/s41181-019-0073-4

**Published:** 2019-09-18

**Authors:** Carlotta Taddei, Victor W. Pike

**Affiliations:** Molecular Imaging Branch, National Institute of Mental Health, National Institutes of Health, 10 Center Drive, Rm B3C342, Bethesda, MD 20892-1003 USA

**Keywords:** Carbon-11, PET, Carbon monoxide, Radiochemistry, Carbonylation, Radiotracer

## Abstract

[^11^C]Carbon monoxide is an appealing synthon for introducing carbon-11 at a carbonyl position (C=O) in a wide variety of chemotypes (e.g., amides, ketones, acids, esters, and ureas). The prevalence of the carbonyl group in drug molecules and the present-day broad versatility of carbonylation reactions have led to an upsurge in the production of this synthon and in its application to PET radiotracer development. This review focuses on the major advances of the past 15 years.

## Background

Carbon-11 is an unstable isotope that has a short half-life of 20.4 min. This radioisotope decays to stable boron-11 predominantly by positron emission (99.79%) and to a very low extent by electron capture (0.21%). Replacement of a carbon atom in an organic compound (e.g., drug or biomolecule) with carbon-11 does not modify its biological or physicochemical properties to any appreciable extent (Pike 1997; Scott 2009). These are attractive features for using carbon-11 to develop radiotracers for application with the highly sensitive molecular imaging technique of positron emission tomography (PET).

The short half-life of carbon-11 allows more than one PET experiment in a single day and in the same subject (Antoni 2015), enhancing the speed of data collection and the throughput of subjects. The possibility to perform more than one study in 1 day in the same subject facilitates test-retest studies and comparison of baseline PET data with PET data after a pharmacological intervention (e.g., blocking studies). Consequently, carbon-11 has a uniquely valuable role in expanding the biomedical and clinical applications of PET. However, radiolabeling with carbon-11 requires quick and efficient methods to maximize radiotracer yields (Långström et al. 2007, Dahl et al. 2017a).

Carbon-11 can be produced in high activity, commonly up to about 100 GBq, and with a high molar activity (ratio of radioactivity to mass; *A*_m_) often in a range of 40 to 750 GBq/μmol at the end of synthesis (Gómez-Vallejo et al. 2012) but also up to 9.7 TBq/μmol (Kihlberg et al. 2002; Noguchi and Suzuki 2003; Zhang and Suzuki 2005). By comparison, a single dose of a PET radiotracer for an experiment in a human subject is about 400 MBq. In our experience, a radiotracer *A*_m_ that is greater than 40 GBq/μmol is acceptable for most PET experiments (Dahl et al. 2018). Arbitrarily, the following qualitative description scale for *A*_m_ (GBq/μmol) values has been used in this review: 5–50 GBq/μmol: low; 50–100 GBq/μmol: moderate; 100–200 GBq/μmol: good; 200–400 GBq/μmol: high; 400 GBq/μmol or above: very high.

Carbon-11 is generally produced with a cyclotron by proton bombardment of nitrogen gas according to the ^14^N(p,α)^11^C nuclear reaction. Bombardment in the presence of oxygen (0.5–1%) or hydrogen (5–10%) gives [^11^C]carbon dioxide or [^11^C]methane, respectively. Oxygen or hydrogen can be present after [^11^C]carbon dioxide and [^11^C]methane production, respectively. Oxygen and potential radioactive impurities (e.g., oxygen-15) can be removed by concentrating the cyclotron-produced [^11^C]carbon dioxide in a cryogenic trap (at liquid nitrogen or argon temperature) or over activated molecular sieves, placed after the output of the cyclotron target chamber. Hydrogen can be eliminated by trapping the cyclotron-produced [^11^C]methane in a Porapak column cooled to liquid nitrogen temperature (Landais and Finn 1989). Furthermore, it is necessary to consider possible traces of water in the target gas and in any subsequently used inert delivery gas (e.g., helium). These traces can affect the outcome and reproducibility of subsequent radiolabeling reactions but can be eliminated with a phosphorous pentoxide trap positioned before a reaction apparatus (Landais and Finn 1989).

**Fig. 1:**
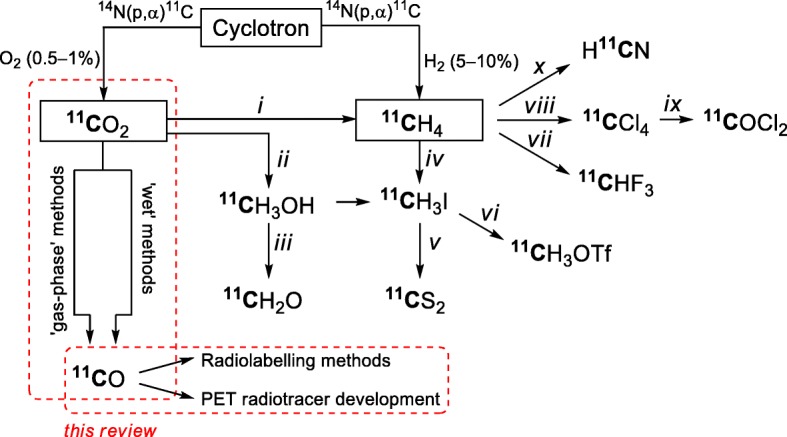
Some established [^11^C]synthons. Reaction conditions: *i*) H_2_/Ni at 400 °C; *ii*) 1. LiAlH_4_, 2. H_2_O; *iii*) Ag at ~ 350 °C; *iv*) I_2_ at 700–725 °C; *v*) S_8_/sand at 500 °C or P_2_S_5_/sand at 400 °C; *vi*) AgOTf at 160–200 °C; *vii*) CoF_3_ at 270 °C; *viii*) CuCl_2_ on pumice at 380 °C; *ix*) 2% O_2_, Fe at 300 °C; *x*) NH_3_/Pt at 920 °C

Several secondary [^11^C]synthons have been developed from the primary cyclotron-produced [^11^C]precursors, [^11^C]carbon dioxide and [^11^C]methane (Fig. 1), with yet others emerging. These [^11^C]synthons enable quick, efficient, versatile, and creative ^11^C-labeling of functionalized molecules (Miller et al. 2008; Rotstein et al. 2013; Taddei and Gee 2018) and underpin fast progress in new PET radiotracer development. Examples of recently developed [^11^C]synthons are [^11^C]carbon disulfide for labeling organosulfur compounds (Haywood et al. 2015; Haywood et al. 2018) and [^11^C]fluoroform (Haskali and Pike 2017) for labeling trifluoromethyl compounds.

[^11^C]Carbon monoxide was early described (Clark and Buckingham 1971) but its emergence as a useful radiochemical synthon was relatively slow to follow. Interest has been accelerated by major developments in mainstream transition metal-mediated carbonylation chemistry and by improvements in [^11^C]carbon monoxide radiosynthesis and application. This review summarizes the progress in [^11^C]carbon monoxide radiochemistry over the past 15 years, and covers advances in production, uses in radiolabeling, and PET radiotracer development (Fig. 1).

### Advances in [^11^C]carbon monoxide production

#### ‘Gas-phase’ [^11^C]carbon monoxide production

**Fig. 2:**
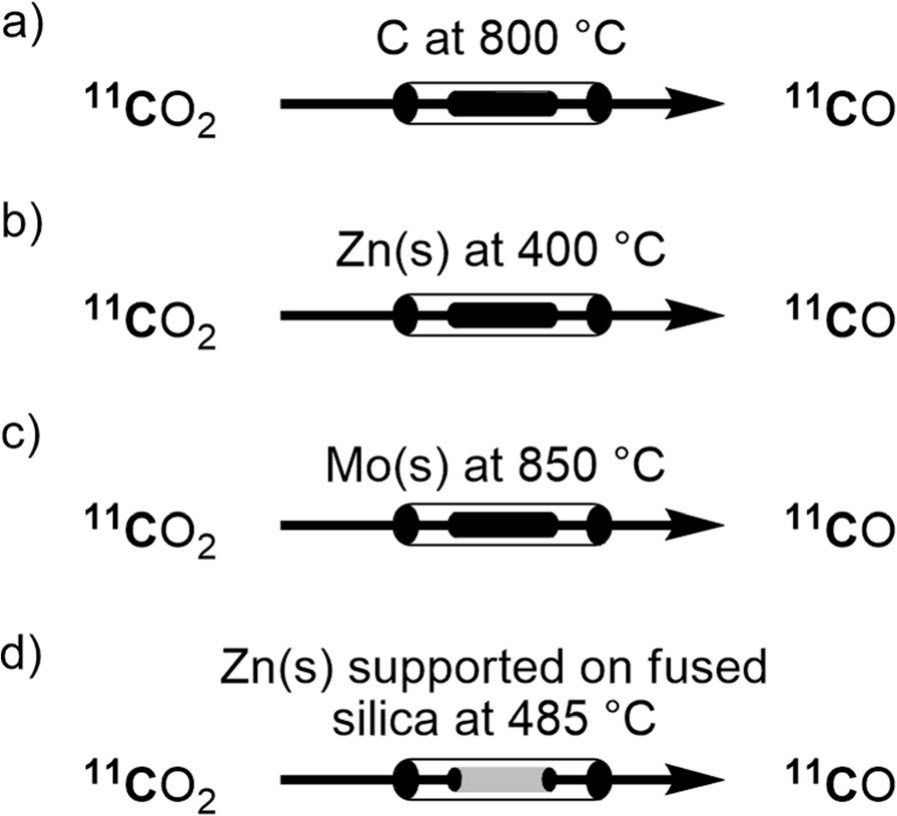
‘Gas-phase’ [^11^C]carbon dioxide reduction on heated columns of: **a** activated charcoal; **b** Zn; **c** Mo; **d** Zn supported on fused silica

The earliest methods for producing [^11^C]carbon monoxide were based on ‘gas-phase’ reduction of cyclotron-produced [^11^C]carbon dioxide over activated charcoal (Fig. 2, a) or a metal surface, such as zinc or molybdenum, at a high temperature (Fig. 2, b and c). The method using activated charcoal (Clark and Buckingham 1971) results in low *A*_m_. Because PET radiotracers commonly need to be produced at high *A*_m_ for efficacy and/or safety, this method is nowadays more or less obsolete.

The first system for routine production of [^11^C]carbon monoxide used a heated zinc column (390–400 °C) (Andersson and Långström 1995; Lidström et al. 1997). The use of pre-concentrated [^11^C]carbon dioxide and a recirculation unit for the reduction produced up to 70% yield and high *A*_m_ typically in the 50 to 500 GBq/μmol range (Lidström et al. 1997). The generated [^11^C]carbon monoxide was utilized to produce ^11^C-labeled ketones in 36–62% isolated yields. In some settings, this method has provided near quantitative yields over many repeated runs (Dahl et al. 2015a). However, this method requires regular column maintenance and its operational success depends on the quality of the zinc. Without adequate maintenance, yields become irreproducible due to formation of zinc oxides on the metal surface over successive heating cycles. Performance may also vary unpredictably with each batch of zinc. Furthermore, the temperature required for reducing [^11^C]carbon dioxide (400 °C) is close to the melting point of zinc (420 °C). Therefore, accidental overheating of the zinc column must be avoided.

With the aim of developing a more convenient, efficient, and reproducible [^11^C]carbon monoxide synthesis, molybdenum heated to 850 °C was introduced as an alternative reductant (Zeisler et al. 1997). [^11^C]Carbon monoxide was obtained in up to 80% yield and with high *A*_m_ (up to 555 GBq/μmol), corrected to the end of radioisotope production (ERP). The produced [^11^C]carbon monoxide was used to synthesize [^11^C]benzophenone with a non-isolated yield of up to 81%. A molybdenum column, although needing to be heated to a much higher temperature, requires less and easier maintenance than a zinc column, and gives acceptably high and more reproducible yields (up to 71%) (Dahl et al. 2015a). These features have allowed the molybdenum method to be widely adopted for the production of [^11^C]carbon monoxide.

Recently, the use of solid-supported zinc has been reported for improving the ‘gas-phase’ reduction of [^11^C]carbon dioxide to [^11^C]carbon monoxide (Dahl et al. 2017b). Molecular sieves, fused silica (originally in the form of silica gel), and molybdenum were investigated as solid supports with fused silica proving to be preferred. The use of a heated column of zinc supported on fused silica at 485 °C (Fig. 2, d) gave an impressive yield (93 ± 3%; *n* = 20). This approach overcame the main limitations of fast deactivation and potential zinc metal melting, experienced with the traditional heated zinc column. The generated [^11^C]carbon monoxide was tested in a ^11^C-carbonylation reaction yielding the corresponding ^11^C-labeled product in 72% yield but with a rather low *A*_m_ of 11 GBq/μmol (Dahl et al. 2017b). Developments to improve the *A*_m_ from this method are needed for regular radiotracer synthesis applications. Nonetheless, this advance signifies an attractive direction for developing effective ‘gas-phase’ [^11^C]carbon monoxide production methods.

A common requirement of these ‘gas-phase’ production methods is for fixed dedicated equipment to occupy valuable hot cell space on a long-term basis. Therefore, alternative ‘wet’ methods for [^11^C]carbon monoxide synthesis using portable apparatus have been sought, as follows.

#### ‘Wet’ [^11^C]carbon monoxide production

Over the past 15 years, many ‘liquid-phase’ methods to produce [^11^C]carbon monoxide have been developed as alternatives to the ‘gas-phase’ methods. These methods aim to avoid the dedicated equipment needs of the ‘gas-phase’ methodologies and to further amplify the utility of [^11^C]carbon monoxide in PET radiotracer development. Examples of these ‘wet’ methods are: i) the decomposition of [^11^C]formyl chloride, ii) the decomposition of [^11^C]silacarboxylic acids, iii) the treatment of [^11^C]carbon dioxide with fluoride-activated disilanes, and iv) electrochemical reduction of [^11^C]carbon dioxide.

**Fig. 3:**
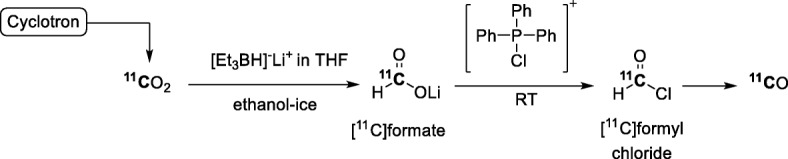
‘Wet’ [^11^C]carbon monoxide production through decomposition of [^11^C]formyl chloride

The production of [^11^C]carbon monoxide from [^11^C]formyl chloride was one of the first ‘wet’ methods to be described (Roeda et al. 2004). This method requires two chemical steps: 1) synthesis of [^11^C]formate (or [^11^C]formic acid) through coupling of [^11^C]carbon dioxide with lithium triethylborohydride in tetrahydrofuran (THF) at low temperature (ethanol-ice bath), and 2) reaction of the [^11^C]formate with a complex formed from hexachloroacetone and triphenylphosphine in THF at room temperature (RT) to give [^11^C]formyl chloride. The [^11^C]formyl chloride decomposes instantly to [^11^C]carbon monoxide (Fig. 3). With this methodology, [^11^C]carbon monoxide has been obtained in a very high yield (98%) but with a low *A*_m_ of 9.3 GBq/μmol. Limitations of this method may be the low reproducibility of the high yield and the low *A*_m_ if the system is not well maintained under an inert atmosphere. Low *A*_m_ may especially arise from the contamination of prepared reagents with atmospheric carbon dioxide, especially the reactive lithium triethylborohydride. Hence, this method has not gained much traction for routine use.

**Fig. 4:**
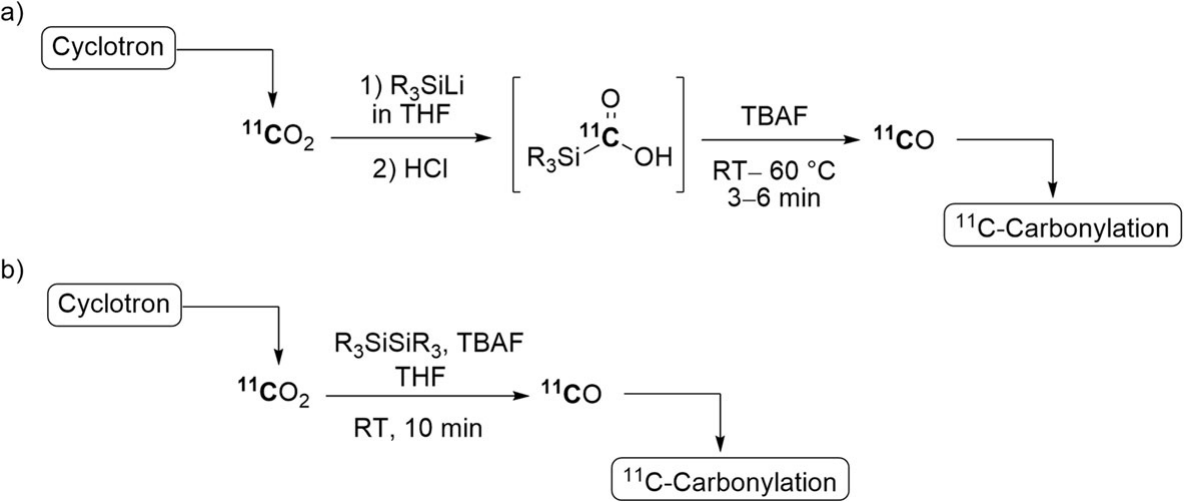
‘Wet’ [^11^C]carbon monoxide production: **a** through decomposition of [^11^C]silacarboxylic acids; **b** through fluoride-activated disilanes

In non-radiochemical studies, silacarboxylic acids have been shown to degrade upon heating or in the presence of a base or a fluoride ion source to eliminate carbon monoxide (Brook 1955; Brook and Gilman 1955; Friis et al. 2011). Based on these studies, [^11^C]silacarboxylic acids have been explored as precursors to [^11^C]carbon monoxide. Lithiosilanes, prepared freshly from chlorosilanes and lithium in THF stirred at RT (either for 3 h or overnight), were found to react with [^11^C]carbon dioxide to yield the corresponding [^11^C]silacarboxylic acids. Specific [^11^C]silacarboxylates and [^11^C]silacarboxylic acids were shown to release [^11^C]carbon monoxide almost quantitatively within a few minutes, upon addition of tetrabutylammonium fluoride (TBAF) solution either at RT or with gentle heating (60 °C) (Fig. 4, a). The generated [^11^C]carbon monoxide can be transferred to a second vial with helium and used in a ^11^C-carbonylation reaction. Many radiolabeled compounds, mainly [^11^C]amides and [^11^C]esters, were produced in this manner. Moderate yields (> 30%) and *A*_m_ in the 70–499 GBq/μmol range were achieved (Taddei et al. 2015; Nordeman et al. 2015; Bongarzone et al. 2017).

The use of lithiosilanes enables [^11^C]carbon monoxide to be produced in a simple two-vial set-up under mild reaction conditions. This overcomes the need for dedicated and fixed infrastructure in the conventional ‘gas-phase’ methods. However, limitations of this methodology are the rather lengthy preparation of fresh lithiosilane before [^11^C]carbon dioxide production, reagent instability, and reactivity with atmospheric carbon dioxide. These can negatively affect the [^11^C]carbon monoxide yield and *A*_m_. Furthermore, the requirement to add the TBAF solution after [^11^C]carbon dioxide capture may also be a limitation of this methodology in a routine setting.

Disilanes have been found to react with carbon dioxide in the presence of a fluoride ion source to yield the corresponding disiloxane with the elimination of carbon monoxide (Lescot et al. 2014). Disilanes are now considered to be compounds that are able to produce [^11^C]carbon monoxide while overcoming some of the constraints of the [^11^C]silacarboxylic acids methodology. 1,2-Dimethyl-1,1,2,2-tetraphenyldisilane was initially chosen for method development. Different fluoride ion sources and solvents were explored leading to TBAF and THF as the optimal activator and reaction medium for the process, respectively. Use of 0.1 equivalent of TBAF was found to be optimal for maximal [^11^C]carbon monoxide production. The [^11^C]carbon monoxide yield increased from 32% to 59% by optimizing the flow rate of the carrier gas for the delivery of [^11^C]carbon dioxide into the reaction system.

Other disilanes have also been investigated. 1,1,2,2-Tetramethyl-1,2-diphenyldisilane gave a maximal [^11^C]carbon monoxide yield of up to 74% at RT within 10 min from ERP (Fig. 4, b). The generated [^11^C]carbon monoxide was used in ^11^C-carbonylation reactions yielding radiolabeled products in high yield and with an *A*_m_ in the 100–120 GBq/μmol range (Taddei et al. 2017a). For optimal *A*_m_ and reproducible yield, an inert atmosphere (e.g., from a helium flow) must be maintained in the reaction vial during reagent preparation and delivery of [^11^C]carbon dioxide to minimize contamination from atmospheric carbon dioxide. This methodology enables the production of [^11^C]carbon monoxide with commercially available compounds under very mild reaction conditions and in a simple apparatus. It also eliminates: 1) the requirement for devoted infrastructure, as in the routinely used oven-based methods (Zn and Mo), and 2) time-consuming reagent preparation, as in the [^11^C]silacarboxylic acids method.

**Fig. 5:**
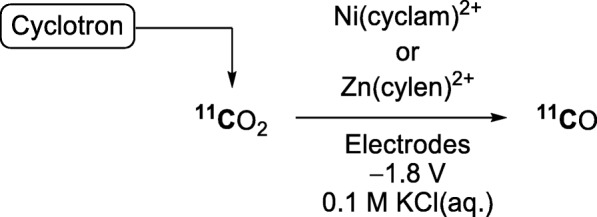
‘Wet’ [^11^C]carbon monoxide production through electrochemical reduction with Ni and Zn complexes

The first electrochemical method for producing [^11^C]carbon monoxide from [^11^C]carbon dioxide was recently described (Anders et al. 2017). This gave [^11^C]carbon monoxide in ≤10% yield when using electrodes with an applied potential of − 1.8 V and a Ni(cyclam)^2+^ or Zn(cyclen)^2+^ complex as an electrocatalyst under aqueous conditions (0.1 M KCl) at 20 °C (Fig. 5). One limitation of this method was the low [^11^C]carbon dioxide trapping efficiency. Therefore, base addition was explored for increasing the retention of [^11^C]carbon dioxide. 1,8-Diazabicyclo[5.4.0]undec-7-ene (DBU) or triethanolamine (TEA) raised the trapping efficiency to 80% but decreased the yield of [^11^C]carbon monoxide to about 3%. This decrease was attributed to an increase in the pH of the solution after the addition of base. Nevertheless, the produced [^11^C]carbon monoxide was used in a test carbonylation reaction and gave the ^11^C-labeled product with an *A*_m_ of 56 GBq/μmol. Further development is needed for this method to become attractive and widespread for regular [^11^C]carbon monoxide production. Especially, a substantial improvement in yield is required.

#### Advances in trapping and utilization of [^11^C]carbon monoxide

Major obstacles that must be surmounted for effective utilization of gaseous [^11^C]carbon monoxide are the generally low solubility and retention of this gas in organic solvents. Various types of apparatus have been introduced to address this issue. A system in which gaseous [^11^C]carbon monoxide is recirculated through the reactor was the first to be introduced (Lidström et al. 1997). The introduction of high-pressure miniature autoclaves quickly followed (Kihlberg and Långström 1999; Hostetler and Burns 2002; Kihlberg et al. 2006). If needed, these could be operated at high temperature (~ 200 °C). Such autoclaves are still in use. A high-pressure (~ 3.5 MPa) apparatus was reported to trap [^11^C]carbon monoxide in organic solvents with high efficiency (generally > 90%) allowing the ^11^C-labeling of various chemotypes in good yields (> 37%) (Dahl et al. 2015a). Moreover, loop reactors and micro-autoclaves enable efficient [^11^C]carbon monoxide utilization because the volume ratio of gas-phase to solution-phase within the reactor can be kept small (Eriksson et al. 2004, 2006, 2007).

A further notable advance has been the use of xenon to carry [^11^C]carbon monoxide efficiently into reaction media. Xenon is highly soluble in organic solvents, such as THF (Gibanel et al. 1993), and its use as delivery gas avoids undesirable build-up of pressure in the reaction vessel (Eriksson et al. 2012).

**Fig. 6:**
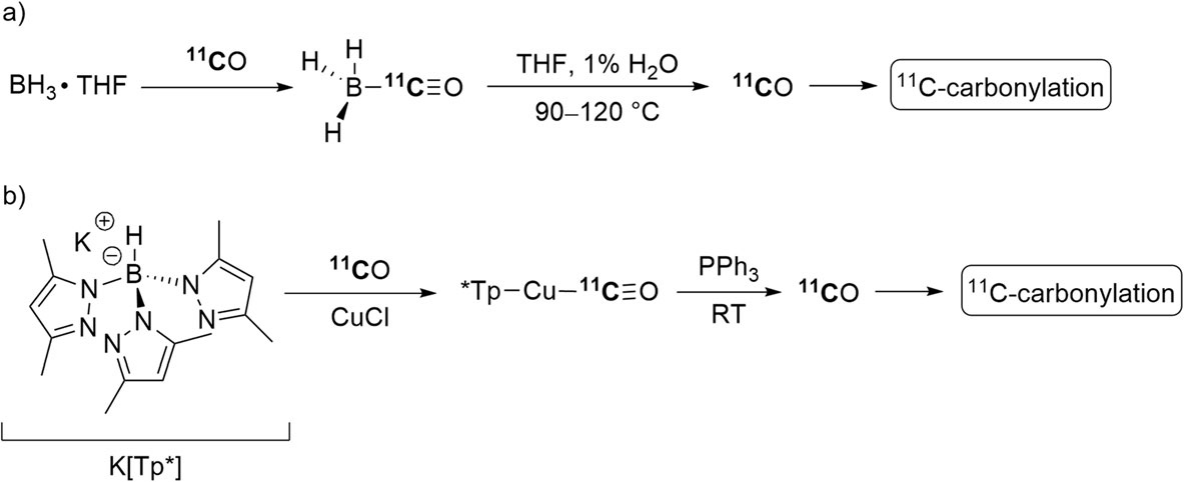
[^11^C]Carbon monoxide trapping and release: **a** from borane-THF complex; **b** from copper(I) scorpionate

In addition to the development of improved synthesis apparatus, several compounds able to trap and then release [^11^C]carbon monoxide under specific conditions, such as borane-THF and copper complexes, have been used to improve the retention and utilization of [^11^C]carbon monoxide in solution. [^11^C]Boron carbonyl complexes, produced by reaction of [^11^C]carbon monoxide with a borane-THF complex in THF, have been found to release the [^11^C]carbon monoxide upon heating (Fig. 6, a) (Audrain et al. 2004). This source of concentrated [^11^C]carbon monoxide was used in a Pd-mediated ^11^C-aminocarbonylation of iodobenzene to give [^11^C]*N*-benzylbenzamide in up to 47% yield and the Pd-mediated ^11^C-carbonylation of 2-bromobenzyl alcohol to give [^11^C]isobenzofuran-1(3*H*)-one in up to 27% yield from trapped [^11^C]carbon monoxide. Trapping efficiency was high (> 90%) under the reported conditions.

Another example of a complex able to trap and release [^11^C]carbon monoxide is copper(I) scorpionate (copper(I) tris(pyrazolyl)borate, Cu(Tp^*^)). This can be formed from commercially available reagents and traps [^11^C]carbon monoxide from carrier streams with very high efficiency (96%). Release of [^11^C]carbon monoxide from this complex is also highly efficient (99%) in the presence of a competing ligand, such as triphenylphosphine (Fig. 6, b) (Kealey et al. 2009, 2014a, 2014b). The released [^11^C]carbon monoxide was used for ^11^C-carbonylation reactions in situ to give substituted [^11^C]amides and [^11^C]ureas in moderate to high yields (≥ 47%).

### Advances in radiolabeling methods with [^11^C]carbon monoxide

**Fig. 7:**
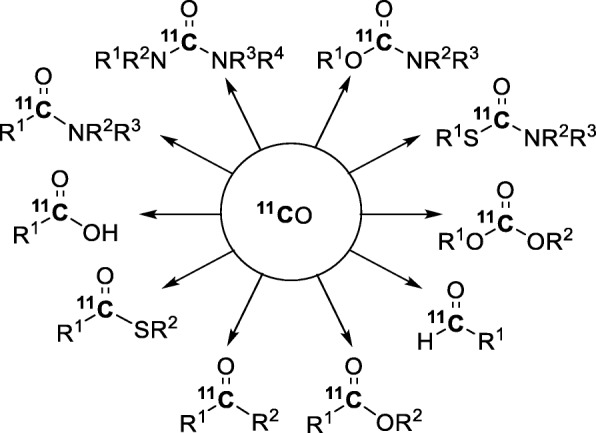
Versatility of ^11^C-carbonylation reactions with [^11^C]carbon monoxide

[^11^C]Carbon monoxide is an appealing synthon for labeling a vast array of radiotracer chemotypes (Fig. 7) because of the broad versatility of modern transition-metal mediated carbonylation reactions (Brennführer et al. 2009; Gadge and Bhanage 2014; Cornilleau et al. 2015; Nielsen et al. 2018). Over the past 15 years, the radiochemistry of [^11^C]carbon monoxide has been widely investigated and expanded (Kealey et al. 2014b; Rahman 2015; Rotstein et al. 2016). Herein are summarized some of the latest advances in the use of [^11^C]carbon monoxide in Pd-mediated reactions, UV-promoted reactions, and other transition metal-mediated reactions for the synthesis of a vast variety of [*carbonyl*-^11^C]compounds (e.g., ^11^C-labeled ureas, amides, esters).

**Fig. 8:**
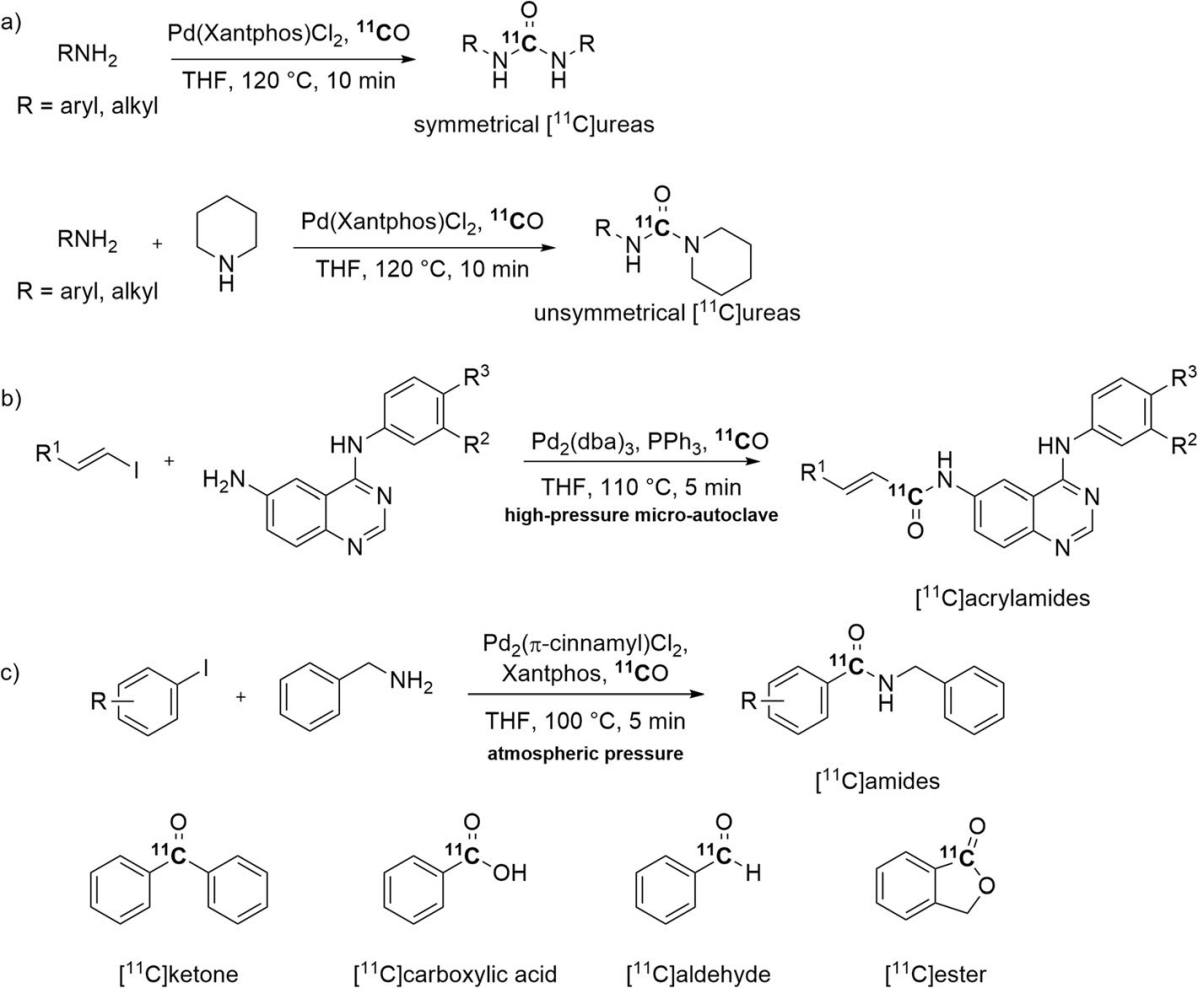
Pd-Mediated ^11^C-carbonylations producing **a** [^11^C]ureas; **b** [^11^C]acrylamides; **c** [^11^C]amides, [^11^C]esters, [^11^C]carboxylic acids, an [^11^C]aldehyde, and a [^11^C]ketone

[^11^C]Carbon monoxide has been recognized as a potentially attractive alternative to [^11^C]phosgene for the synthesis of [^11^C]ureas. Although [^11^C]phosgene is highly reactive and has been widely used for [^11^C]ureas synthesis (Roeda and Dollé 2010), the regular production of this [^11^C]synthon is very challenging and tedious, and often provides low *A*_m_. Pd(II)-Mediated oxidative ^11^C-carbonylation reactions between aliphatic or aromatic amines with [^11^C]carbon monoxide have been explored recently for the synthesis of symmetrical and unsymmetrical [^11^C]ureas (Roslin et al. 2017) (Fig. 8, a). Xenon was used to carry the [^11^C]carbon monoxide into a septum-sealed glass reactor. [^11^C]Ureas were obtained in yields up to 61% and with high *A*_m_ in the 247–319 GBq/μmol range, under quite mild conditions (≤ 120 °C, 10 min).

Pd-Mediated carbonylation reactions have featured in the ^11^C-carbonylation of several other chemotypes. Functionalized [^11^C]acrylamides have been prepared through Pd(0)-mediated carbonyl insertions between 4-anilino-6-aminoquinazoline and substituted vinyl iodides in a high-pressure micro-autoclave (Fig. 8, b). The desired [^11^C]acrylamides were obtained in 24–61% yields and with an *A*_m_ of 60 GBq/μmol (Åberg and Långström 2012).

In another study, Pd(II)-mediated reactions were used to synthesize [^11^C]amides, a [^11^C]ester, a [^11^C]carboxylic acid, a [^11^C]aldehyde, and a [^11^C]ketone at atmospheric pressure under thermal heating (Fig. 8, c) (Dahl et al. 2013). The catalyst, Pd_2_(π-cinnamyl)Cl_2,_ was paired with the ligand Xantphos. This ligand performed better than others, probably because of its wide bite angle. Microwave heating improved the yields in the reactions of electron-deficient aryl halides. Furthermore, this methodology has also been used with aryl chlorides as substrates to produce an [^11^C]arylcarboxylic acid and [^11^C]aryl esters (Dahl et al. 2014).

**Fig. 9:**
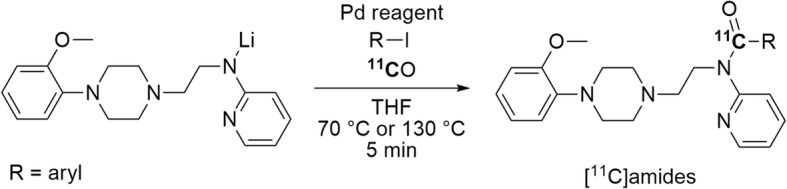
Synthesis of [^11^C]amides from lithiated amines through Pd-mediated ^11^C-carbonylation

Another approach for producing [^11^C]amides is a quick (5 min) one- or two-pot Pd-mediated ^11^C-carbonylation reaction between an aryl halide and a lithiated amine that has been freshly prepared from amine and *n-*butyllithium. Eleven [^11^C]amides were obtained in isolated yields of 18–72% (Fig. 9) (Itsenko et al. 2007). This method extends the scope of Pd-mediated ^11^C-carbonylation reactions to the use of weakly nucleophilic amines.

**Fig. 10:**
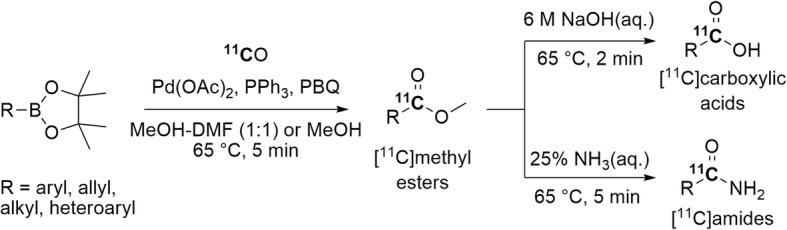
Synthesis of [^11^C]methyl esters through Pd(0)-mediated ^11^C-carbonylation of boronic acid pinacol esters. PBQ = *p-*benzoquinone

^11^C-Carbonylation of aryl, heteroaryl, allyl, and alkyl boronic acid pinacol esters in the presence of *p-*benzoquinone and triphenylphosphine has been investigated for producing a variety of [^11^C]methyl esters. Yields in the 6–80% range were obtained from quick reactions (5 min) performed under atmospheric pressure with gentle heating (Fig. 10) (Ishii et al. 2015). The conversion of [^11^C]methyl esters into the corresponding [^11^C]carboxylic acids or [^11^C]amides was easily achieved through treatment with sodium hydroxide or aqueous ammonium, respectively (Fig. 10). This approach was also applied to the synthesis of [*carbonyl*-^11^C]aspirin and [*carbonyl*-^11^C]salicylic acid in yields of 15 ± 2% and 58 ± 5%, respectively. These yields surpass those obtained via the direct ^11^C-carboxylation of Grignard reagents (Sasaki et al. 1999).

**Fig. 11:**
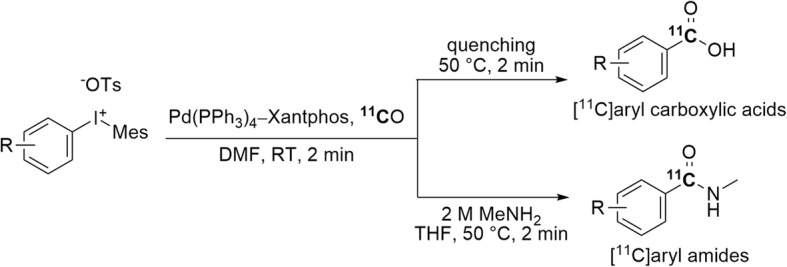
Syntheses of [^11^C]aryl carboxylic acids and [^11^C]aryl amides through ^11^C-carbonylation of diaryliodonium salts

Diaryliodonium salts have featured prominently as reactive precursors for radiohalogenation reactions (Pike 2018; Telu et al. 2019). They are also known to undergo Pd(II)-mediated carbonyl insertion with [^11^C]carbon monoxide (Al-Qahtani and Pike 2000). A two-pot procedure for the rapid Pd(0)-mediated ^11^C-carbonylation of aryl(mesityl)iodonium salts at RT and low pressure has been recently developed (Fig. 11) (Altomonte et al. 2017). A range of non-mesityl [^11^C]aryl carboxylic acids and [^11^C]aryl amides bearing an electron-withdrawing or electron-donating group were obtained in up to 71% yield within 2 min at RT.

**Fig. 12:**
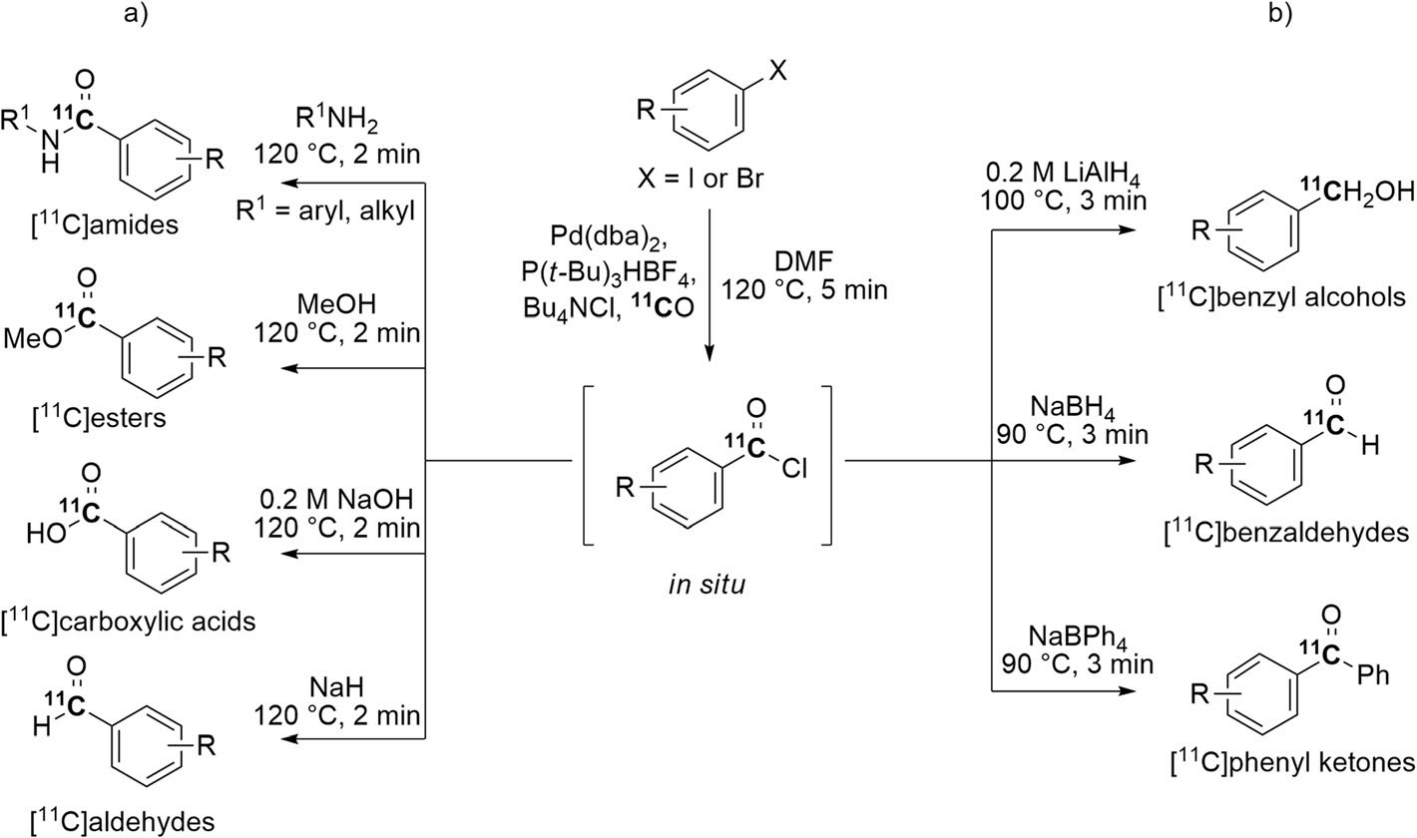
Two-step methodology for the synthesis of **a** [^11^C]amides, [^11^C]esters, [^11^C]carboxylic acids, and [^11^C]aldehydes; **b** [^11^C]benzyl alcohols, [^11^C]benzaldehydes, and [^11^C]phenyl ketones via [^11^C]benzoyl acid chlorides

[^11^C]Amides, [^11^C]esters, [^11^C]carboxylic acids, and [^11^C]aldehydes have also been prepared via a two-step methodology, composed of: 1) in situ generation of a [^11^C]benzoyl acid chloride through a Pd(0)-mediated ^11^C-carbonylation of an aryl iodide, and 2) subsequent reaction of the generated [^11^C]benzoyl acid chloride with a chosen nucleophile in a second reaction vial (Fig. 12, a) (Dahl and Nordeman 2017). Many diverse ^11^C-labeled products were obtained within 7 min in 41–93% yields. This methodology has been successfully extended to the synthesis of [^11^C]benzyl alcohols, [^11^C]benzaldehydes, and [^11^C]phenyl ketones (Fig. 12, b) (Roslin et al. 2018) and is an attractive alternative to a former method for accessing [^11^C]aryl acid chlorides based on the ^11^C-carboxylation of Grignard reagents (Pike et al. 1982; Krasikova et al. 2009).

**Fig. 13:**
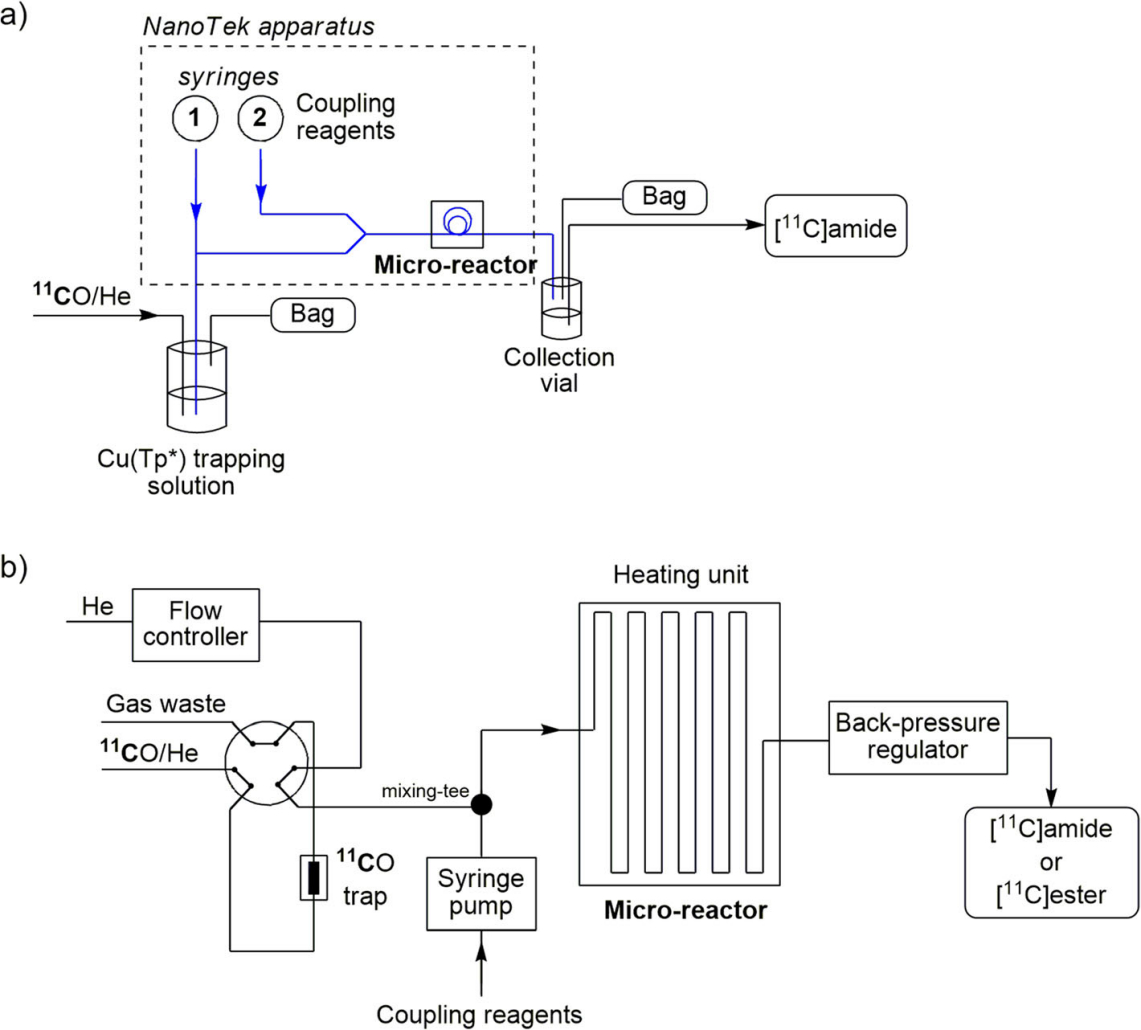
Pd-Mediated syntheses of: **a** [^11^C]amides in a microfluidic reactor; **b** [^11^C]amides and [^11^C]esters in a gas-liquid microfluidic platform

Pd-Mediated ^11^C-carbonylation reactions have been performed in novel experimental set-ups (Fig. 13). A microfluidic reactor with a solution containing [^11^C]carbon monoxide in the form of its complex with copper(I) scorpionate (Cu(Tp^*^)) was applied to the synthesis of a model [^11^C]amide, namely [^11^C]*N-*benzylbenzamide. [^11^C]*N-*Benzylbenzamide was obtained with a radiochemical purity of 69% under a microreactor temperature of 200 °C and with a flow rate of 20 μL/min per syringe, corresponding to a very short residence time (< 1 min) in the microreactor (Fig. 13, a) (Kealey et al. 2011). [^11^C]Dibenzylurea was formed as a significant byproduct (22 ± 1%).

The first application of a gas-liquid segmented-flow microfluidic platform was described for the Pd-mediated synthesis of a variety of ^11^C-labeled compounds (e.g., [^11^C]amides and [^11^C]esters) (Fig. 13, b). The ^11^C-labeled compounds were obtained in good yields (≥ 38%) and with *A*_m_ in the 40–54 GBq/μmol range (Dahl et al. 2015b). These yields supersede those from a previously reported gas-liquid annular flow microfluidic apparatus (Miller et al. 2011). This improvement was attributed to the larger gas-liquid interface in the segmented-flow because of the continuous formation of microbubbles (Dahl et al. 2015b).

**Fig. 14:**
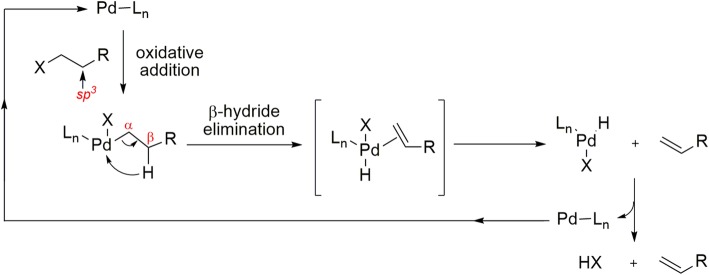
Representation of β-elimination mechanism

**Fig. 15:**
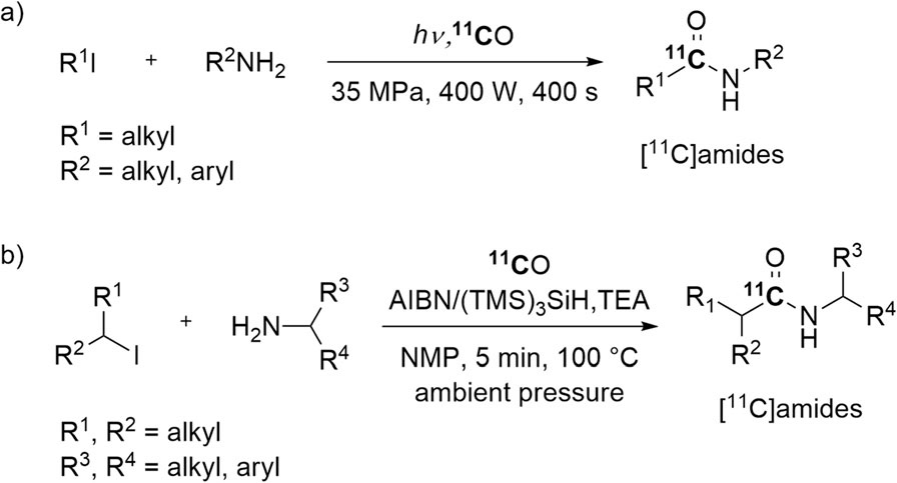
Radical ^11^C-cabonylation of substrates with β-hydrogens: **a** at high pressure; **b** at ambient pressure

Generally, Pd-mediated carbonylation reactions are limited to compounds that lack β-hydrogens on saturated *sp*^3^ carbons, such as aryl halides or triflates. Compounds with β-hydrogens do not produce carbonylated products with carbon monoxide because the β-hydride elimination from σ-alkyl-Pd intermediates is competitive and usually predominant (Fig. 14). To circumvent this issue, a high-pressure reactor with a quartz window open to light from a mercury lamp has been used successfully for radical ^11^C-carbonylations of substrates bearing β-hydrogen atoms (Fig. 15, a) (Itsenko et al. 2004). Radical ^11^C-carbonylation has also been improved recently by using a low-pressure xenon-[^11^C]carbon monoxide delivery unit and azobisisobutyronitrile (AIBN) as a radical initiator under thermal conditions (Fig. 15, b). This approach showed broad substrate compatibility with alkyl iodides containing β-hydrogen atoms. The desired [^11^C]amides were obtained in 9–25% isolated yields and with an *A*_m_ of 101 ± 13 GBq/μmol (Chow et al. 2016).

**Fig. 16:**
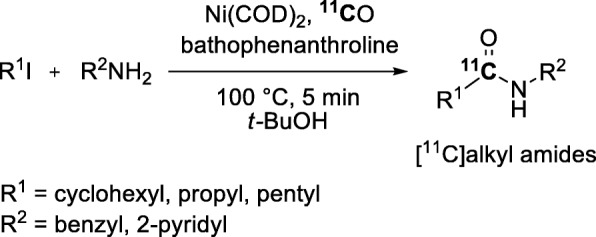
Ni(0)-Mediated cross-coupling reaction of non-activated alkyl iodides bearing a β-hydrogen atom. Ni(COD)_2_ = bis(1,5-cyclooctadiene)nickel(0)

Transition-metals other than palladium have been used to mediate ^11^C-carbonylation reactions. One example is the nickel(0)-mediated ^11^C-carbonylation on non-activated alkyl iodides bearing a β-hydrogen atom (Fig. 16). [^11^C]Alkyl amides were obtained in 33–57% yields within 5 min when using *tert-*butanol as solvent (Rahman et al. 2016). However, this method requires handling of the nickel catalyst under an argon atmosphere to avoid any reagent oxidation that would result in failure of the radiolabeling reaction.

**Fig. 17:**
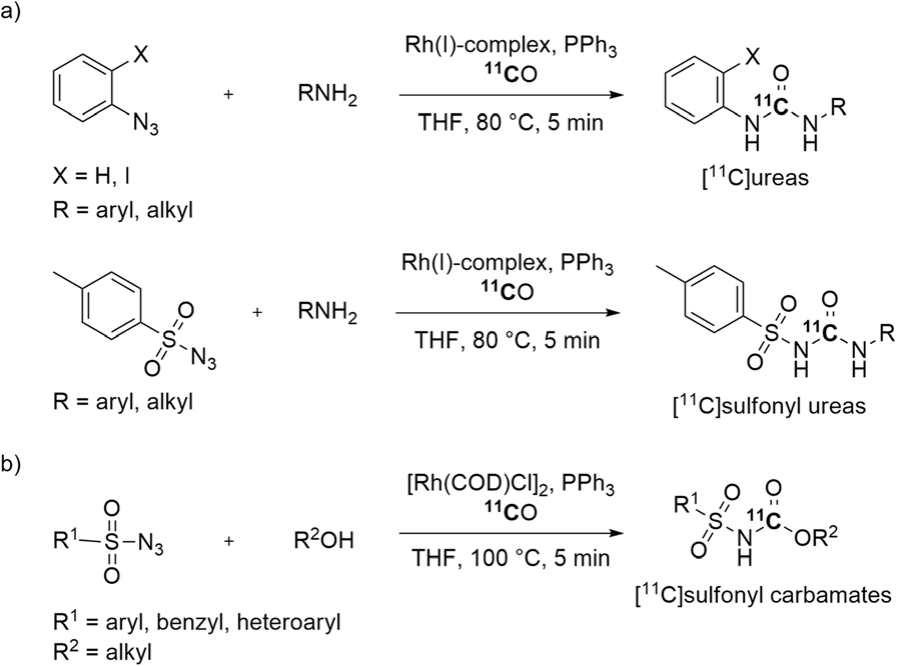
Rh(I)-Mediated ^11^C-carbonylations producing: **a** [^11^C]ureas and [^11^C]sulfonyl ureas; **b** [^11^C]sulfonyl carbamates. [Rh(COD)Cl]_2_ = chloro(1,5-cyclooctadiene)rhodium(I) dimer

Substituted [^11^C]ureas and [^11^C]sulfonyl ureas have been synthesized through rhodium(I)-mediated insertion of [^11^C]carbon monoxide between azides and amines (Fig. 17, a) (Åberg and Långström 2011). This method shows good functional group tolerance. The radiolabeled products were obtained under mild conditions in 14–96% yields and with an *A*_m_ in the 100–600 GBq/μmol range.

A Rh(I)-mediated multicomponent reaction between sulfonyl azides, alcohols, and [^11^C]carbon monoxide has been reported for producing [^11^C]sulfonyl carbamates (Fig. 17, b) (Stevens et al. 2016). This method is compatible with structurally diverse sulfonyl azides. Various [^11^C]sulfonyl carbamates were produced in good yields (33–88%).

### [^11^C]Carbon monoxide for PET radiotracer development

As a result of the major advances that have taken place in [^11^C]carbon monoxide production and radiochemical utility, ^11^C-labeled carbonyl groups have become increasingly prevalent in candidate PET radiotracer designs (Rotstein et al. 2016). The possibility to radiolabel carbonyl groups is of extreme relevance for using PET in drug distribution studies, where the unchanged drug structure needs to be radiolabeled and where motifs, such as radiolabeled methyl groups or fluorine atoms, cannot be introduced.

Although many applications of [^11^C]carbon monoxide require the presence of transition metals (e.g.*,* Pd) as reagents for ^11^C-carbonylations, such metals have not been an issue for producing radiotracers under CGMP conditions. For example, [^11^C]UCB-J and [^11^C]FPEB have been produced for human use through Pd-mediated labeling reactions (Lohith et al. 2014; Nabulsi et al. 2016; Lohith et al. 2017; DiFilippo et al. 2019). In our laboratory, we have found that normal methods for radiotracer purification (e.g., HPLC) are capable of reducing Pd residues to sub-ppb levels (e.g., for the [^11^C]FPEB synthesis (Lohith et al. 2014)). These levels are well below the limits considered acceptable for parental administration of non-radiopharmaceutical drugs (1 ppm per day; (USP 2017).

Additional considerations pertain to ^11^C-carbonylation reactions for PET radiotracer synthesis, such as steric constraints adjacent to carbonylation sites, functional group tolerance in more structurally elaborate substrates, and ease of product purification. Moreover, the overall efficiency of the production process of a PET radiotracer, from cyclotron radioisotope production to the radiochemically pure product ready for intravenous injection, is also an important aspect to consider. Ideally, the process should be readily amenable to automation. Henceforth, we summarize the progress that has been made towards producing PET radiotracers through reactions with [^11^C]carbon monoxide.

**Fig. 18:**
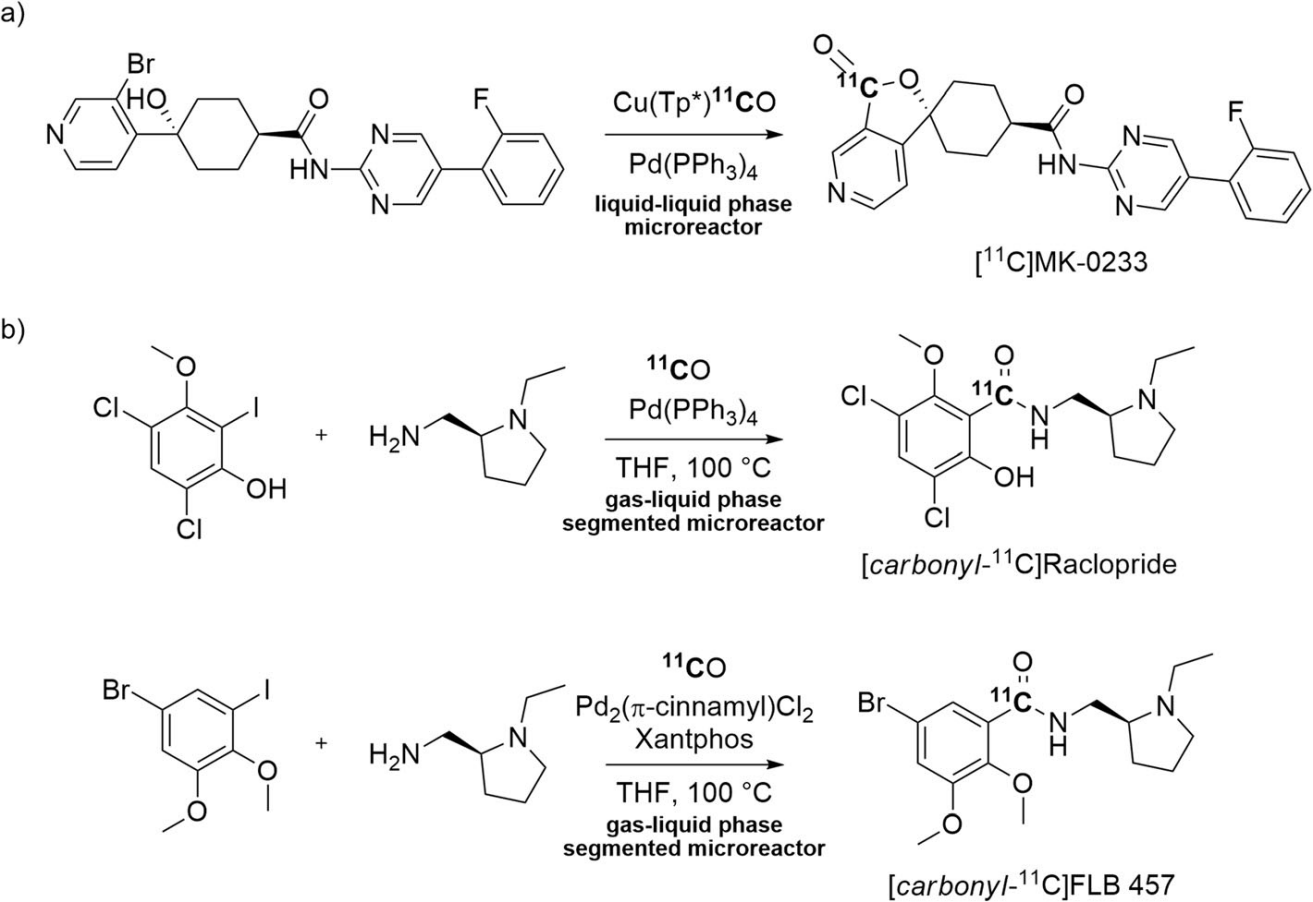
Synthesis of **a** [^11^C]MK-0233; **b** [*carbonyl*-^11^C]raclopride, and [*carbonyl*-^11^C]FLB 457

The microfluidic apparatus described earlier has been tested for the syntheses of PET radiotracers. The liquid-liquid phase microreactor featuring the copper complex (Cu(Tp^*^)), was implemented to synthesize a ^11^C-labeled neuropeptide Y Y5 receptor antagonist, [^11^C]MK-0233 (Table 1, Entry 1). [^11^C]MK-0233 was produced through a Pd-mediated ^11^C-carbonylation reaction with [^11^C]carbon monoxide. [^11^C]Carbon monoxide was released from a Cu(Tp^*^)^**11**^**C**O complex for an internal cyclization reaction to give [^11^C]MK-0233 (Fig. 18, a). [^11^C]MK-0233 was obtained ready for intravenous injection in a yield of 7.1 ± 2.2% from utilized [^11^C]carbon monoxide and with an *A*_m_ of 100 ± 15 GBq/μmol at 27 min from ERP (Kealey et al. 2011).

**Table 1:** Radiotracers radiolabeled using [^11^C]carbon monoxide, the corresponding biological target, and disease relevance

Entry	Radiotracer	Biological target	Disease relevance	Reference(s)
1		Y Y5 receptor	Weight loss	(Kealey et al. 2011)
2		D_2_ receptor	Movement disorders, schizophrenia	(Dahl et al. 2015b), (Rahman et al. 2015), (Andersen et al. 2015)
3		D_2_ receptor	Movement disorders, schizophrenia	(Dahl et al. 2015b)
4		TSPO	Neuroinflammation	(Rahman and Långström 2007)
5		CB_1_	Neuropsychiatric disorders (*e.g.*, schizophrenia, anxiety, and depression)	(Donohue et al. 2008)
6		mGluR1	Anxiety, mood disorders, stroke, epilepsy, pain, and schizophrenia	(Hong et al. 2015)
7		HDAC6	Neurodegenerative disorders and cancer	(Lu et al. 2016)
8		BACE-1	Alzheimer’s disease	(Nordeman et al. 2014)
9		5-HT_1B_	Migraine, depression, and anxiety	(Lindberg et al. 2019)
10		OGA	Alzheimer’s disease	^a^
11		H_3_R	Neuropsychiatric and neurodegenerative disorders	(Dahl et al. 2013), (Dahl et al. 2018)
12		H_3_R	Neuropsychiatric and neurodegenerative disorders	(Siméon et al. 2017)
13		Serine/threonine kinase	Melanomas	(Slobbe et al. 2017)
14		sHE	Inflammation and neuropathic pain	(Roslin et al. 2017)
15		TG2	Celiac disease, cancer, fibrosis, and neurodegenerative diseases (*e.g.*, Alzheimer’s, Huntington’s, Parkinson’s diseases)	(van der Wildt et al. 2016)
16		GAD	Neuropsychiatric disease (*e.g.*, schizophrenia)	(Taddei et al. 2017b)b
17		cPLA2α	Oxidative stress and neuroinflammation	(Fisher et al. 2018)
18		Functionalized [^11^C]synthon for potential PET radiotracers (*e.g.*, barbiturates)		(Barletta et al. 2006)
19		AT_2_R	Prostate cancer and idiopathic pulmonary fibrosis	(Stevens et al. 2016)
20		Efflux transporter P-gp	Neurological disease (*e.g.*, Alzheimer’s disease)	(Verbeek et al. 2012)
21		TKI	Cancer	(Poot et al. 2013)
22		5-HT_1B/1D_	Locomotion and anxiety	(Lindhe et al. 2011)

^a^Lu S, Haskali MB, Ruley KM, Dreyfus NJ-F, DuBois SL, Soumen P, *et al.* Discovery and development of ^18^F- and ^11^C-labeled LSN3316612 as positron emission tomography radioligands for quantifying O-linked-β-*N*-acetyl-glucosamine hydrolase in brain, *submitted*. ^b^Taddei C, Filp U, Pekošak A, Poot AJ, Windhorst AD, Gee AD. Synthesis of a ^11^C-tracer for potential brain glutamic acid decarboxylase (GAD) targeting, *submitted*

[*O-methyl*-^11^C]Raclopride and [*O-methyl*-^11^C]FLB 457 are well known radiotracers for PET imaging of human striatal and extrastriatal D_2_/D_3_ receptors, respectively (Ito et al. 1999; Okubo et al. 1999). [*Carbonyl*-^11^C]raclopride and [*carbonyl*-^11^C]FLB 457 (Entries 2 and 3) have been synthesized in a gas-liquid phase segmented microreactor through Pd-mediated ^11^C-carbonylation reactions (Fig. 18, b). High non-isolated yields of [*carbonyl*-^11^C]raclopride and [*carbonyl*-^11^C]FLB 457 (79 ± 1% and 61 ± 4%, respectively) were obtained from utilized [^11^C]carbon monoxide (Dahl et al. 2015b). A series of model [*carbonyl*-^11^C]amides were produced similarly in the same study and had moderate *A*_m_ (40–54 GBq/μmol).

[*carbonyl*-^11^C]Raclopride has also been produced through a one-step ^11^C-carbonylation reaction at atmospheric pressure using Pd_2_(π-cinnamyl)Cl_2_ as catalyst and Xantphos as supporting ligand (Rahman et al. 2015). The yield of [*carbonyl*-^11^C]raclopride ready for intravenous injection was 50 ± 5% from [^11^C]carbon monoxide trapped in the reaction vial and the *A*_m_ was 34 GBq/μmol. The trapping efficiency was moderate (65 ± 5%). PET experiments were performed in monkey to compare [*carbonyl*-^11^C]raclopride and [*O*-*methyl-*^11^C]raclopride. A key question in this study was whether the change in radiolabeling position from *O*-methyl to carbonyl would be detrimental to [^11^C]raclopride performance in vivo. However, the PET experiments showed remarkably similar results with regards to protein binding, emergence of radiometabolites in plasma, and quantitative outcome measures of D_2_/D_3_ receptors (Rahman et al. 2015). This similarity argues against demethylation as being a prime route of radiotracer metabolism; more likely is that amide hydrolysis occurs.

**Fig. 19:**
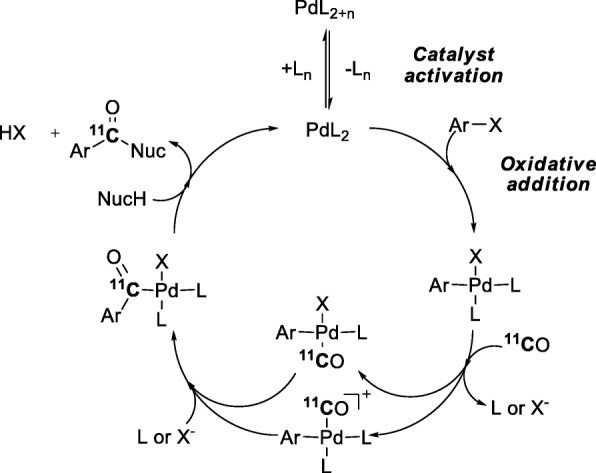
Pd-Mediated ^11^C-carbonylation of aryl halides

**Fig. 20:**
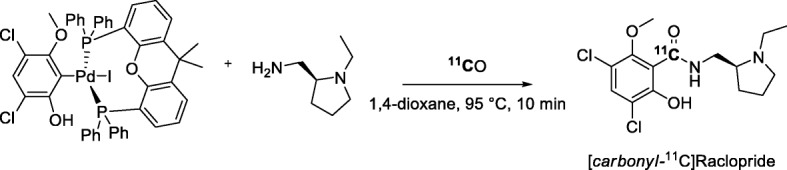
Use of a pre-formed Pd-aryl oxidative addition complex for the synthesis of [*carbonyl*-^11^C]raclopride

It has been reasoned that two potentially slow steps in the Pd-mediated ^11^C-carbonylation of aryl halides (Fig. 19), namely activation of the Pd catalyst and subsequent oxidative addition of the aryl halide, can be beneficially avoided by pre-synthesis and isolation of the desired Pd-aryl oxidative addition complex (Aryl)Pd(X)L_n_ (Andersen et al. 2015). This was confirmed with the ^11^C-aminocarbonylation of an isolated Pd-aryl oxidative addition complex, formed from Pd(dba)_2_ and Xantphos, for the synthesis of [*carbonyl-*^11^C]raclopride (Fig. 20) (Andersen et al. 2015). This radiotracer was obtained in 38–44% yield from trapped [^11^C]carbon monoxide within 8 min from ERP and with a high *A*_m_ in the 333–407 GBq/μmol range. An advantage of this radiosynthetic approach was that it gave [*carbonyl-*^11^C]raclopride in high initial purity (94 ± 1%) thereby reducing separation challenge. Two other potential radiotracers, [*carbonyl*-^11^C]olaparib and a neuropeptide Y Y3 receptor antagonist, [*carbonyl*-^11^C]JNJ 31020028, were also efficiently radiolabeled from pre-formed Pd-complexes.

**Fig. 21:**
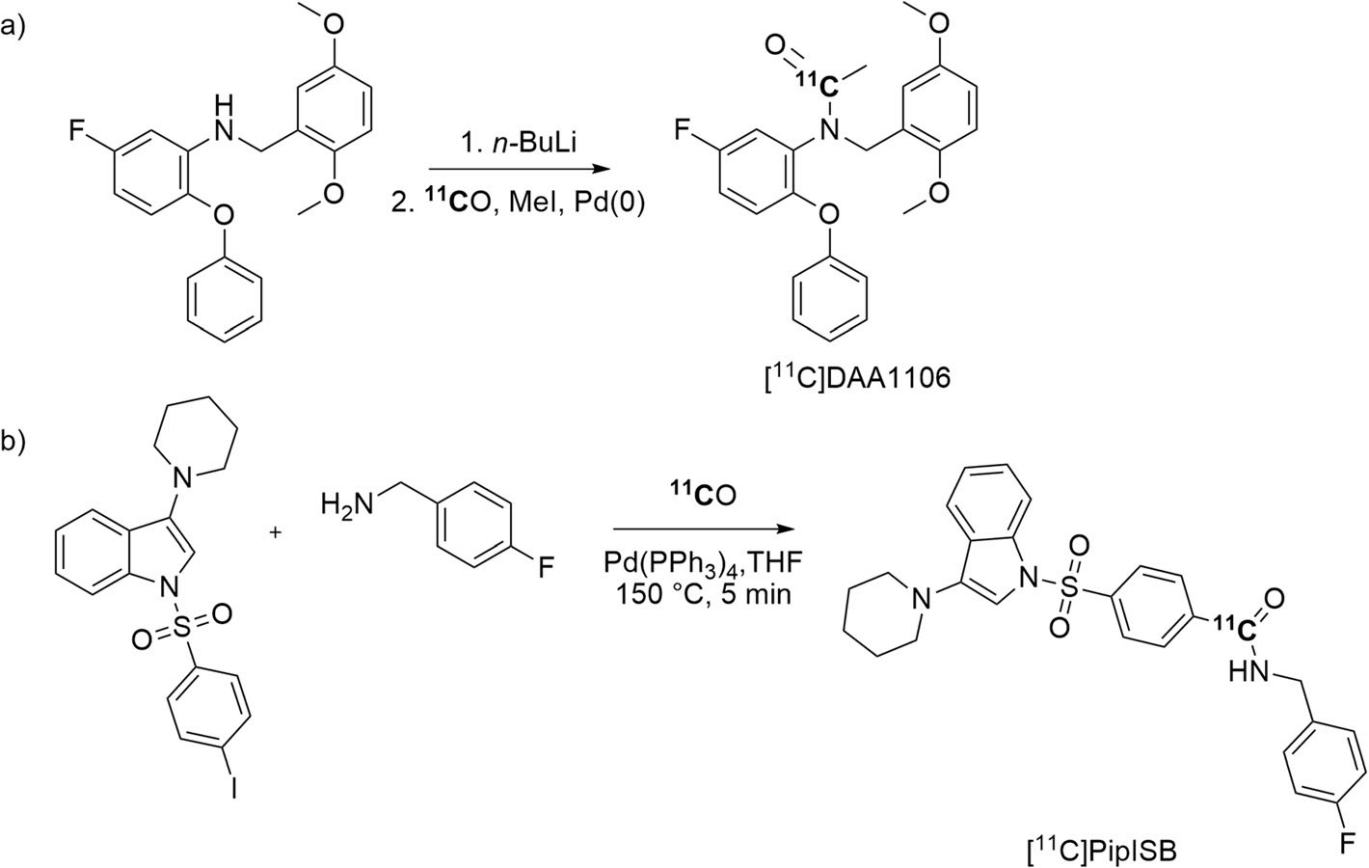
Synthesis of **a** [^11^C]DAA1106 and **b** [^11^C]PipISB via Pd-mediated ^11^C-aminocarbonylation reactions

The synthesis of selective and potent PET radiotracers for translocator protein (TSPO), a biomarker for neuroinflammation, is of continuing major interest (Dupont et al. 2017). The TSPO radioligand, [*O*-*methyl-*^11^C]DAA1106 (Zhang et al. 2003), has found quite wide application in clinical research. The ^11^C-labeling of DAA1106, and some of its analogues has been achieved through Pd-mediated ^11^C-aminocarbonylation reactions. A strong base, such as *n-*butyllithium, was used to activate the amine and improve the overall yields. [*carbonyl*-^11^C]DAA1106 (Entry 4) was produced in 30% yield and with a high *A*_m_ of 455 GBq/μmol at 36 min after ERP (Fig. 21, a) (Rahman and Långström 2007). Analogues were also obtained in moderate yields and with high *A*_m_. Investigations are needed to compare the behavior of [*carbonyl-*^11^C]DAA1106 with that of the established [*O*-*methyl-*^11^C]DAA1106, especially to test whether PET imaging performance might differ because of potential differing radiometabolite profiles.

The development of PET radiotracers for imaging brain cannabinoid subtype-1 (CB_1_) receptors has been pursued because of the implication of this receptor in a range of neuropsychiatric disorders, such as schizophrenia, anxiety, and depression. [^11^C]PipISB, a selective and high affinity CB_1_ receptor radioligand (Entry 5) has been produced ready for intravenous injection within 44 min from ERP through a Pd-mediated ^11^C-aminocarbonylation reaction between an aryl iodide precursor and [^11^C]carbon monoxide in overall yields of 3–12% from [^11^C]carbon monoxide and with an *A*_m_ in the 21–67 GBq/μmol range (Fig. 21, b) (Donohue et al. 2008). This radiotracer was studied and compared with the ^18^F-labeled version in monkey. Each radiotracer gave high proportions of CB_1_ specific binding in brain (Finnema et al. 2009). However, neither [^11^C]PipISB nor [^18^F]PipISB has been advanced to human study because other higher-performing radiotracers for PET imaging of human brain CB_1_ receptors appeared concurrently (e.g., [^11^C]MePPEP (Terry et al. 2009) and [^18^F]FMPEP-*d*_2_ (Terry et al. 2010)).

**Fig. 22:**
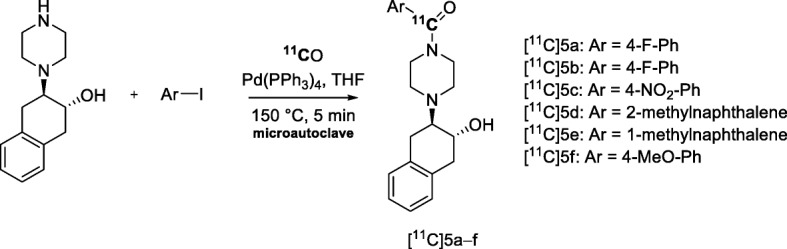
^11^C-Labeling with [^11^C]carbon monoxide of potential VAChT radiotracers

Changes in brain cholinergic neurons are implicated in several neurodegenerative disorders. Vesicular acetylcholine transporter (VAChT) is considered a biomarker for cholinergic neurons. The need of potential PET radiotracers for VAChT has been addressed in the synthesis of a small library ([^11^C]5a − f, Fig. 22) of potential VAChT radiotracers (Bergman et al. 2014). Six piperazine-based radiotracers were produced through Pd-mediated ^11^C-carbonylation insertions between synthesized amines and commercially available aryl iodides in isolated yields of 4–25% from starting radioactivity and with *A*_m_ in the 124–597 GBq/μmol range. Two of these radiotracers exhibited specific binding to VAChT in vitro. However, these results did not support further evaluation of these radiotracers in pre-clinical PET imaging.

**Fig. 23:**
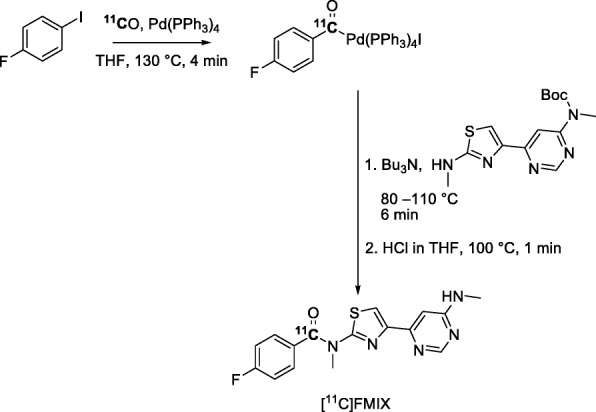
Two-pot synthesis of [^11^C]FIMX

The metabotropic glutamate subtype-1 receptor (mGluR1) has been implicated in anxiety and mood disorders. Therefore, mGluR1 has gained interest as a potential drug target. A Pd-mediated [^11^C]carbon monoxide insertion reaction has been used to produce [^11^C]FIMX as a potential PET radiotracer for imaging brain mGluR1 (Hong et al. 2015). [^11^C]FIMX (Entry 6), was synthesized through a two-pot procedure, composed of Pd-mediated carbonylation of 1-fluoro-4-iodobenzene with [^11^C]carbon monoxide in a first pot and subsequent treatment of the [^11^C]acylpalladium complex with Boc-protected amine precursor followed by Boc removal in a second pot (Fig. 23). [^11^C]FIMX was obtained ready for intravenous injection in an overall yield of about 5% from initial cyclotron-produced radioactivity and with an *A*_m_ of 102 ± 31 GBq/μmol at 42 ± 3 min from ERP. PET imaging in monkey showed [^11^C]FIMX to be an effective radiotracer for mGluR1 and to have imaging properties very similar to those of [^18^F]FIMX (Hong et al. 2015; Xu et al. 2013). A potential advantage of the ^11^C-label over the ^18^F-label is the possibility for two PET measurements in a single subject in 1 day. This possibility is convenient for drug occupancy studies which may require a series of paired baseline and pharmacological challenge experiments in single subjects.

**Fig. 24:**
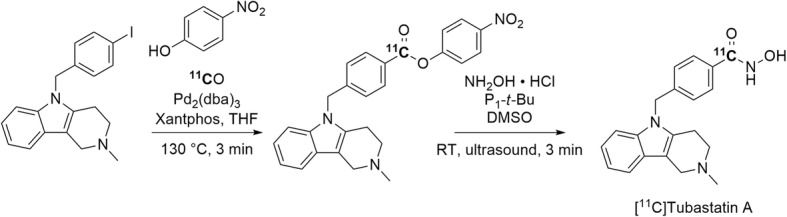
Radiosynthesis of [^11^C]tubastatin A. P_1_-*t*-Bu = *tert*-butylimino-*tris*(dimethylamino)phosphorene

The synthesis of PET radiotracers for histone deacetylase 6 (HDAC6) has gained interest because of the possible involvement of this enzyme in cancer and various neuropsychiatric disorders (Wey et al. 2016). A hydroxamic group features prevalently in many inhibitors of this enzyme. The HDAC6 inhibitor, tubastatin A, has been radiolabeled with carbon-11 (Entry 7) in the hydroxamic acid group via a two-step process (Fig. 24), namely: 1) Pd-mediated [^11^C]carbon monoxide insertion between an aryl iodide and *p-*nitrophenol, and 2) ultrasound-assisted hydroxyaminolysis of the pre-formed [^11^C]ester with excess hydroxylamine in the presence of the strong phosphazene base P_1_-*t*-Bu (Lu et al. 2016). [^11^C]Tubastatin A was obtained ready for intravenous injection in a yield of 16 ± 6% (*n* = 4) from initial cyclotron-produced [^11^C]carbon dioxide and with a low *A*_m_ of 7.4 GBq/μmol at 61 min from ERP. However, attempts to apply this radiolabeling approach to other hydroxamic acids were unsuccessful because the final hydroxyaminolysis step gave only the corresponding [^11^C]carboxylic acids.

**Fig. 25:**
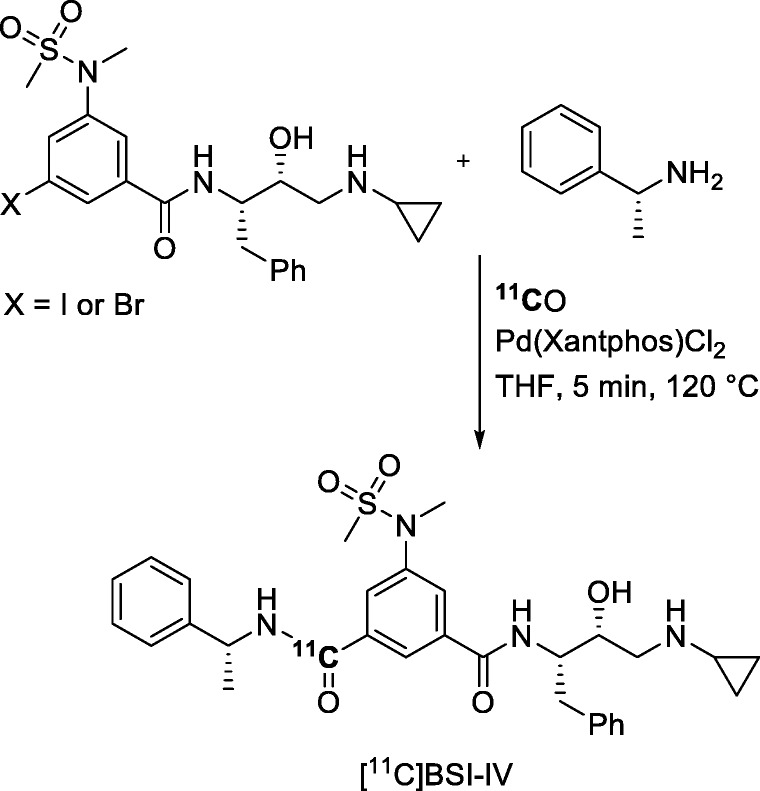
Synthesis of [^11^C]BSI-IV through a Pd-mediated ^11^C-aminocarbonylation reaction

Novel PET radiotracers for the diagnosis and monitoring of Alzheimer’s disease are a constant demand in the PET neuroimaging field. An hydroxyethylamine-based inhibitor for enzyme β-secretase 1 (BACE-1), namely [^11^C]BSI-IV (Entry 8), has been labeled through a Pd-mediated ^11^C-aminocarbonylation reaction on an aryl halide precursor (Fig. 25) as a potential useful PET radiotracer for evaluating Alzheimer’s disease in vivo. [^11^C]BSI-IV was produced in isolated yields of 29 ± 12% (*n* = 12) from trapped [^11^C]carbon monoxide and with a high *A*_m_ of 790 ± 155 GBq/μmol (Nordeman et al. 2014). Trapping efficiency was about 60%. Biodistribution and PET-CT studies were performed on healthy rat brain sections and male rats, respectively. However, these studies showed low specific binding of [^11^C]BSI-IV to BACE-1 in vitro, fast clearance in vivo, and low uptake in brain. These results proved [^11^C]BSI-IV to be an unpromising PET radiotracer for BACE-1.

**Fig. 26:**
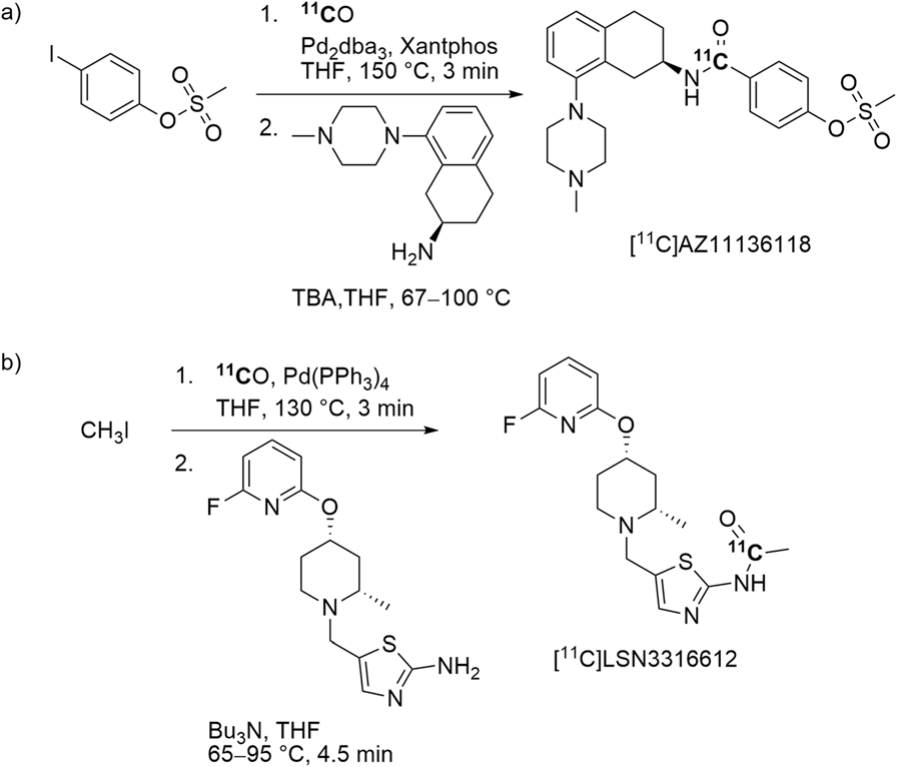
^11^C-Labeling of **a** [^11^C]AZ11136118 and **b** [^11^C]LSN3316612 with [^11^C]carbon monoxide

The serotonin subtype 1B (5-HT_1B_) receptor is involved in migraine, depression, and anxiety. [^11^C]Carbon monoxide has been implemented for radiolabeling AZ11136118, a high-affinity full 5-HT_1B_ receptor agonist. [^11^C]AZ11136118 (Entry 9) was obtained ready for intravenous injection in 6.4 ± 1.6% overall yield from starting [^11^C]carbon dioxide and with an *A*_m_ of 83 ± 51 GBq/μmol (*n* = 7) within 50 min from ERP through a two-pot Pd-mediated ^11^C-carbonylation reaction (Fig. 26, a) (Lindberg et al. 2019). In this study, another high-affinity full 5-HT_1B_ receptor agonist, [^11^C]AZ11895987, was synthesized using [^11^C]methyl triflate for an *N*-methylation reaction. PET imaging with the two radiotracers were performed in monkeys to investigate how the intrinsic activity of full 5-HT_1B_ receptor agonist radiotracers might affect PET imaging outcomes. However, both [^11^C]AZ11136118 and [^11^C]AZ11895987 exhibited too low brain uptake to be useful PET radiotracers (Lindberg et al. 2019).

Very recently Lu et al. presented the development of effective radiotracers for PET imaging of the enzyme *O-*GlcNAcase (OGA), a potential biomarker and therapeutic target for tauopathy (Lu S, Haskali MB, Ruley KM, Dreyfus NJ-F, DuBois SL, Soumen P, et al. Discovery and development of ^18^F- and ^11^C-labeled LSN3316612 as positron emission tomography radioligands for quantifying O-linked-β-N-acetyl-glucosamine hydrolase in brain, submitted). [^3^H]LSN3316612 had shown high selectivity and potency for binding to OGA in post mortem brains of rat, monkey, and human. The radiotracer, [^11^C]LSN3316612 (Entry 10), was produced for intravenous injection with a two-step procedure through Pd-mediated ^11^C-carbonylation reaction (Fig. 26, b) in a low overall yield (3.5 ± 1.3%) from cyclotron-produced [^11^C]carbon dioxide and with an *A*_m_ of 74 ± 39 GBq/μmol at 50 min after ERP. Evaluation of [^11^C]LSN3316612 with PET in monkey demonstrated that this radiotracer is effective for quantifying OGA in monkey brain.

**Fig. 27:**
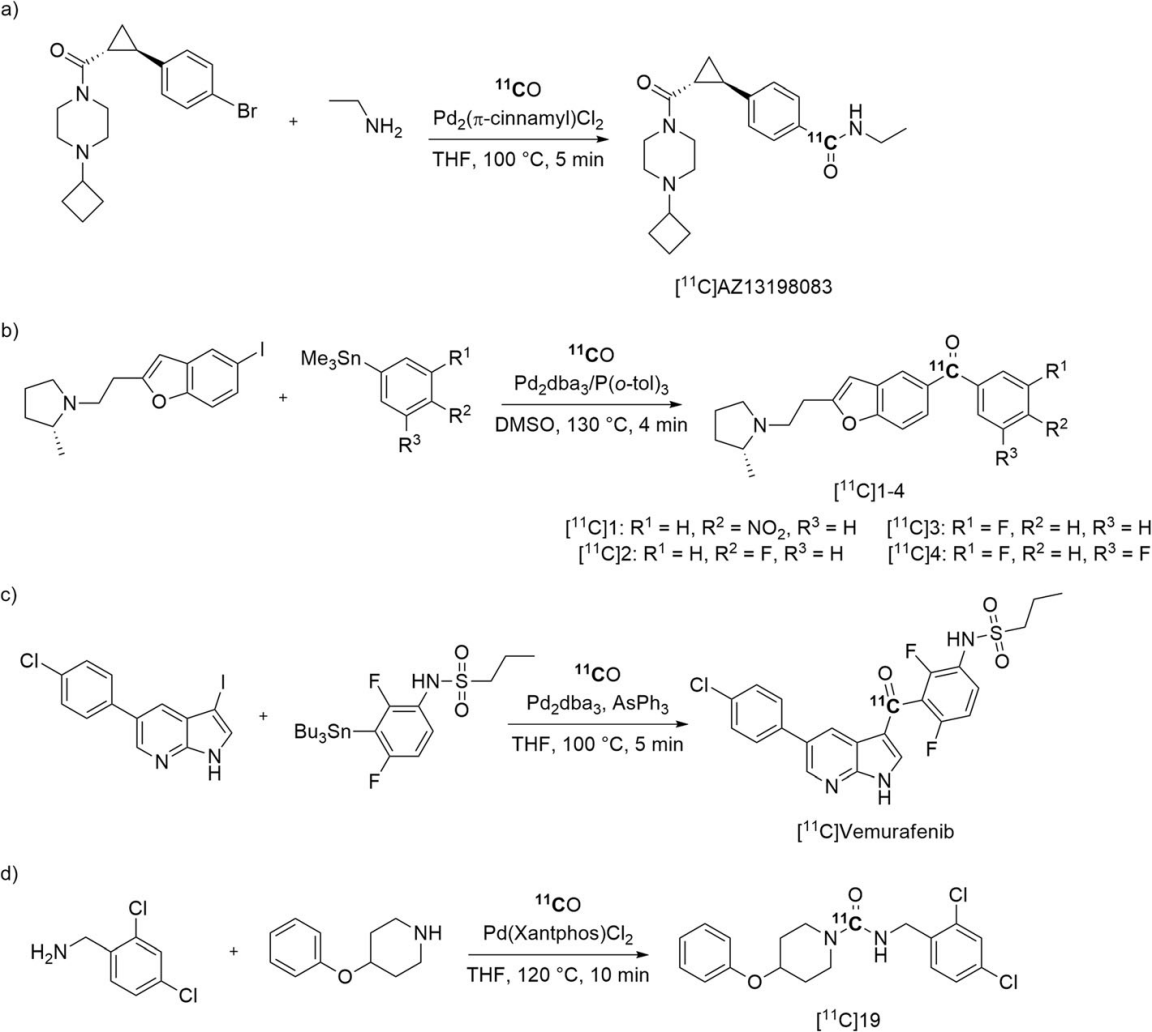
Synthesis of potential H_3_R radiotracers: **a** [^11^C]AZ13198083; **b** [^11^C]1–4; **c** ^11^C-labeling of [^11^C]vemurafenib; and **d** of an sHE inhibitor [^11^C]19 through Pd-mediated ^11^C-carbonylation reactions

The histamine type-3 receptor (H_3_R) has been considered a drug target for treating neuropsychiatric disorders. Novel amide ligands for H_3_R, have been radiolabeled through Pd-mediated ^11^C-aminocarbonylation reactions and evaluated with PET in monkeys (Dahl et al. 2018). [*carbonyl*-^11^C]AZ13153556, [*carbonyl*-^11^C]AZD5213, and [*carbonyl*-^11^C]AZ13198083 were obtained in good non-isolated yields (≥ 80%) from starting [^11^C]carbon monoxide and with a moderate *A*_m_ in the 19–28 GBq/μmol range at time of intravenous injection (Dahl et al. 2018). [*carbonyl*-^11^C]AZ13198083 (Entry 11) (Fig. 27, a) had first been reported in 2013 (Dahl et al. 2013). In the later study, [*carbonyl*-^11^C]AZ13198083 was found to be the most promising of this candidate triad for potential PET imaging of H_3_R in living human subjects.

Structurally elaborate [^11^C]aryl ketones, [^11^C]1–4 (Entry 12) (Fig. 27, b), have been produced through Pd-mediated [^11^C]carbon monoxide insertion reactions between aryl iodides and aryltributylstannanes as other examples of potential PET radiotracers for imaging brain H_3_R. [^11^C]1–4 were obtained for pre-clinical use in 5–9% yields from cyclotron-produced [^11^C]carbon dioxide and with *A*_m_ ≥ 115 GBq/μmol (Siméon et al. 2017). However, these radiotracers were not advanced to evaluation with PET.

Recently, vemurafenib (Entry 13), a serine/threonine kinase inhibitor, has been radiolabeled with carbon-11 to assess its potential as a PET radiotracer for tumors expressing the BRAF^V600E^ mutation. Labeling was achieved through Pd-mediated ^11^C-carbonylation in 21 ± 4% yield from [^11^C]carbon monoxide and with an *A*_m_ of 55 ± 18 GBq/μmol (Fig. 27, c) (Slobbe et al. 2017). However, preliminary in vitro and biodistribution studies in mice suggested that [^11^C]vemurafenib is not a suitable PET radiotracer for identifying the BRAF^V600E^ mutation in vivo (Slobbe et al. 2017).

The development of PET radiotracers for soluble epoxide hydrolase (sHE), an enzyme implicated in inflammation and neuropathic pain (Shen and Hammock 2012), may be of interest to further understand these pathophysiological processes. Recently, a urea sHE inhibitor, [^11^C]19 (Entry 14), has been radiolabeled through a Pd-mediated oxidative ^11^C-carbonylation between an aryl amine and an aliphatic amine (Fig. 27, d). [^11^C]19 was obtained in 41 ± 7% yield from [^11^C]carbon monoxide and with an *A*_m_ in the 247–319 GBq/μmol range at 41–43 min from ERP (Roslin et al. 2017). However, evaluation of this radiotracer with PET imaging has not been reported.

**Fig. 28:**
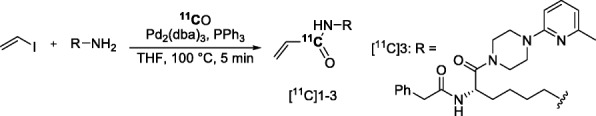
Pd-Mediated ^11^C-carbonylation approach used to ^11^C-label potential TG2 inhibitors

Pd-Mediated ^11^C-aminocarbonylation reactions with [^11^C]carbon monoxide have been exploited to generate substituted [^11^C]acrylamides as candidate PET radiotracers for active tissue transglutaminase (TG2) (Fig. 28) (van der Wildt et al. 2016). Three ^11^C-labeled inhibitors ([^11^C]1–3) for TG2 were produced in 38–55% isolated yields from starting [^11^C]carbon monoxide and with *A*_m_ ≥ 202 GBq/μmol. Ex vivo biodistribution and plasma stability studies were performed in healthy Wistar rats. One of the radiotracers, [^11^C]3 (Entry 15), showed high metabolic stability, low brain uptake, and selective binding to TG2 in tumor sections (van der Wildt et al. 2016).

**Fig. 29:**
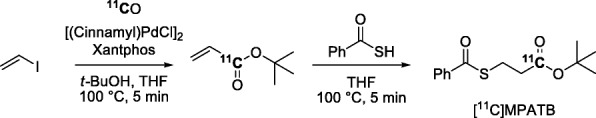
Synthesis of [^11^C]MPATB through Pd-mediated ^11^C-carbonylation followed by a thiol-ene reaction

Glutamic acid decarboxylase (GAD) is the major enzyme responsible for the synthesis of the neurotransmitter γ-aminobutyric acid (Fenalti et al. 2007) and dysregulation of GAD function has been implicated in neuropsychiatric disorders, such as schizophrenia (Akbarian and Huang 2006). Attempts have been made to develop PET radiotracers for GAD to potentially elucidate its function in neuropsychiatric disorders. [^11^C]MPATB (Entry 16), a ^11^C-labeled analogue of the GAD inhibitor 3-mercaptopropionic acid, has been synthesized through a one-pot Pd-mediated ^11^C-carbonylation on vinyl iodide in the presence of *tert-*butanol and subsequent thiol-ene reaction with thiobenzoic acid (Fig. 29). [^11^C]MPATB was produced in high radiochemical purity of 99% and an isolated yield of 0.5–3% from starting [^11^C]carbon monoxide (Taddei et al. 2017b; Taddei C, Filp U, Pekošak A, Poot AJ, Windhorst AD, Gee AD. Synthesis of a ^11^C-tracer for potential brain glutamic acid decarboxylase (GAD) targeting, submitted). Further development to improve the overall yield of [^11^C]MPATB may be required.

**Fig. 30:**
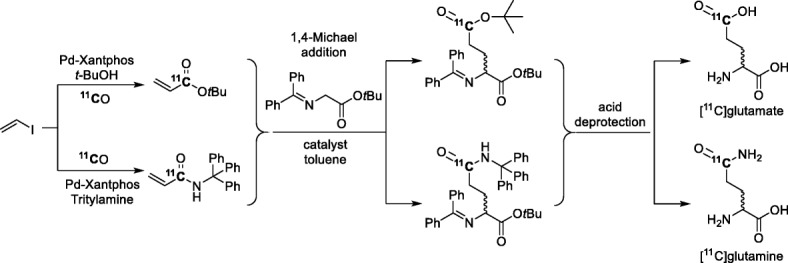
Synthesis of [^11^C]glutamate and [^11^C]glutamine via Pd-mediated ^11^C-carbonylation followed by Michael addition reactions

[^11^C]Carbon monoxide has also been used with vinyl iodide to produce racemic side-chain labeled ^11^C-labeled amino acids, namely the neurotransmitter glutamate and the essential amino acid glutamine through a three-step approach: 1) Pd-mediated ^11^C-carbonylation of vinyl iodide in the presence of *tert-*butanol or tritylamine, followed by 2) Michael addition reactions with the generated [^11^C]acrylate and [^11^C]acrylamide, respectively, and finally 3) acid hydrolysis (Fig. 30) (Filp et al. 2017). The initial ^11^C-carbonylation reactions proceeded in high yields under preferred conditions (> 70%). The Michael addition reaction was moderately efficient when using CsOH·H_2_O as base for the synthesis of [^11^C]glutamine. Furthermore, introduction of a chiral phase-transfer catalyst resulted in some stereoselectivity for the Michael addition reaction.

**Fig. 31:**
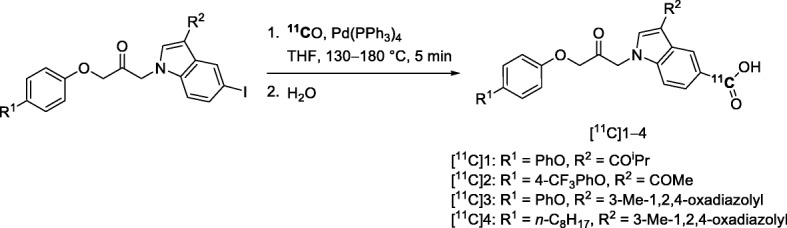
^11^C-Labeling approach used for four candidate inhibitors of cPLA2α

Four carboxylic acid-type high-affinity inhibitors, [^11^C]1–4 (Entry 17), of cytosolic phospholipase A2α (cPLA2α), an enzyme implicated in neuroinflammatory conditions, have been radiolabeled through Pd-mediated ^11^C-carbonylation on aryl iodide precursors followed by hydrolysis (Fig. 31). The desired ^11^C-labeled inhibitors were obtained ready for intravenous injection in 1.1–5.5% overall yields from [^11^C]carbon dioxide and with an *A*_m_ in the 70–141 GBq/μmol range (Fisher et al. 2018). These candidate radiotracers were evaluated with PET imaging in mice. However, they proved to be ineffective PET radiotracers because of their low entry and retention in brain following intravenous administration.

**Fig. 32:**
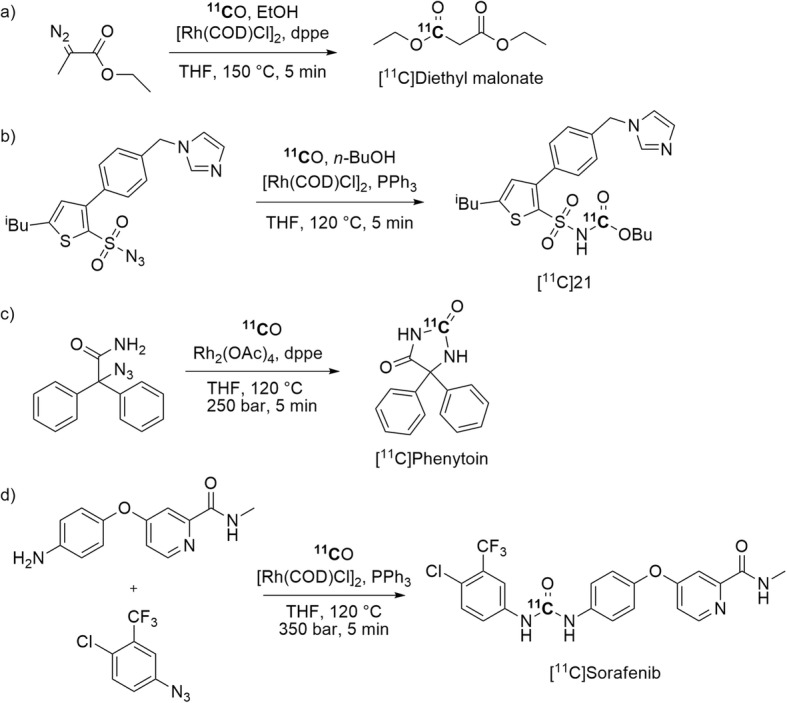
Rh-Mediated synthesis of: **a** [^11^C]diethyl malonate; **b** an agonist of AT_2_R, [^11^C]21; **c** [^11^C]phenytoin; **d** [^11^C]sorafenib

Transition-metals other than palladium have been used to mediate ^11^C-carbonylation reactions for the synthesis of candidate PET radiotracers. One example is the Rh-promoted ^11^C-carbonylation reaction with ethyl diazoacetate and ethanol to produce [*carbonyl-*^11^C]diethyl malonate (Entry 18) in an isolated yield of 20 ± 7% from starting radioactivity and with an *A*_m_ of 127 GBq/μmol at EOS (Fig. 32, a) (Barletta et al. 2006). This difunctional labeling synthon was then alkylated to produce [*carbonyl-*^11^C]diethyl 2,2-diethylmalonate in 50% yield as a candidate PET radiotracer. This approach is promising for the synthesis of different [*carbonyl-*^11^C]malonates as potential PET radiotracers (Barletta et al. 2006).

A Rh-mediated ^11^C-carbonylation reaction has also been used to synthesize a ^11^C-labeled agonist of non-peptide angiotensin II subtype 2 receptor (AT_2_R), a receptor involved in prostate cancer. This radiotracer, namely [^11^C]C21 (Entry 19), was obtained from a sulfonyl azide in a multicomponent reaction in 24 ± 10% yield from [^11^C]carbon monoxide and with an *A*_m_ in the 34–51 GBq/μmol range (Fig. 32, b), and then evaluated with PET in rats (Stevens et al. 2016). [^11^C]C21 bound specifically to prostate tumor cells expressing AT_2_R but showed rapid metabolism and excretion, limiting its use as a PET radiotracer. Nevertheless, these findings provide an impetus for further development of AT_2_R-selective PET radiotracers.

Rh-Promoted ^11^C-carbonylations have also been used to synthesize a candidate radiotracer for the efflux transporter P-glycoprotein (P-gp) in rats, namely [^11^C]phenytoin (Fig. 32, c; Entry 20). [^11^C]Phenytoin was produced ready for intravenous injection in an overall yield of 22 ± 4% and with an *A*_m_ of 277 ± 67 GBq/μmol at the end of synthesis (Verbeek et al. 2012). However, PET evaluation in rats showed [^11^C]phenytoin to be a weak Pg-p substrate. Another example is the Rh-mediated synthesis of [*urea-*^11^C]sorafenib (Fig. 32, d; Entry 21), a ^11^C-labeled tyrosine kinase inhibitor. [*urea-*^11^C]Sorafenib was obtained ready for intravenous injection in a yield of 27% ± 11% and with an *A*_m_ of 30–50 GBq/μmol within 50 min (Poot et al. 2013). [*urea-*^11^C]Sorafenib proved to be stable in in vivo. This may encourage future PET studies for tumor targeting in tumor-bearing mice.

**Fig. 33:**
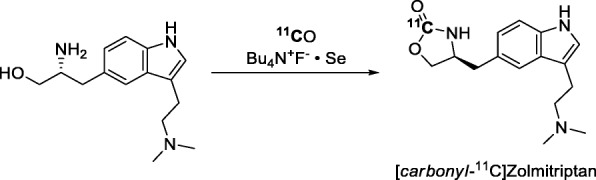
Radiosynthesis of [^11^C]zolmitriptan through Se-mediated ^11^C-carbonylation

Selenium-mediated ^11^C-carbonylation reactions were early described for the synthesis of functionalized [^11^C]carbamates (Kihlberg et al. 2002). More recently, [*carbonyl*-^11^C]Zolmitriptan (Entry 22), a serotonin 5-HT_1B/1D_ receptor agonist, has been prepared in an autoclave through a Se-mediated [^11^C]carbon monoxide insertion reaction (Fig. 33) (Lindhe et al. 2011). [^11^C]Zolmitriptan was used to map binding sites in monkey brain and to characterize the regional distribution of zolmitriptan binding to 5-HT_1_ receptor subtypes. This PET radiotracer showed a high proportion of binding (90%) to high-affinity sites and a discrete regional distribution in monkey brain (Lindhe et al. 2011).

## Conclusions

[^11^C]Carbon monoxide continues to emerge as a useful radiolabeling synthon for introducing the [^11^C]carbonyl group into a wide variety of chemotypes. It can be prepared and utilized efficiently, and high *A*_m_ can be achieved. The radiolabeling reactions with [^11^C]carbon monoxide show high functional group tolerance providing avenues towards attractive single-step PET radiotracer production. Furthermore, the metabolic hydrolysis of PET radiotracers that are ^11^C-labeled in an ester or amide carbonyl site can lead to poorly brain-penetrant radiometabolites, such as the corresponding [^11^C]carboxylic acids. This is especially relevant and useful for avoiding PET signal contamination when PET imaging is performed with radiotracers targeting brain proteins.

Candidate PET radiotracers produced from [^11^C]carbon monoxide have so far advanced only to evaluation in animals. It may be expected that in time some candidate PET radiotracers, synthesized through ^11^C-carbonylation reactions, will justify their production for PET imaging in human subjects. To encourage further progress in this direction, there is a need for commercially available automated radiosynthesis apparatus specific for [^11^C]carbon monoxide production and utility and which provides a high level of consistent performance, especially in a CGMP (current good manufacturing practice) environment.

## Data Availability

Not applicable.

## Abbreviations

5-HT_1B_: 5-Hydroxytryptamine 1B
AIBN: Azobisisobutyronitrile
*A*_m_: Molar activity
AT_2_R: Angiotensin II subtype 2 receptor
BACE-1: β-Secretase 1
BBB: Blood-brain barrier
CB_1_: Cannabinoid type-1
CGMP: Current good manufacturing practice
cPLA2α: Cytosolic phospholipase A2α
DBU: 1,8-Diazabicyclo [5.4.0]undec-7-ene
EOS: End of synthesis
ERP: End of radioisotope production
GAD: Glutamic acid decarboxylase
H_3_R: Histamine type-3 receptor
HDAC6: Histone deacetylase 6
mGluR1: Metabotropic glutamate receptor 1
OGA: *O-*GlcNAcase
PET: Positron emission tomography
P-gp: P-Glycoprotein
RT: Room temperature
sHE: Soluble epoxide hydrolase
TBAF: Tetrabutylammonium fluoride
TEA: Triethanolamine
TG2: Tissue transglutaminase
THF: Tetrahydrofuran
TKI: Tyrosine kinase inhibitor
TSPO: Translocator protein
VAChT: Vesicular acetylcholine transporter
